# Addressing survey fatigue bias in longitudinal social contact studies to improve pandemic preparedness

**DOI:** 10.1038/s41598-025-02235-0

**Published:** 2025-05-23

**Authors:** Shozen Dan, Zhi Ling, Yu Chen, Joshua Tegegne, Veronika K. Jaeger, André Karch, Swapnil Mishra, Oliver Ratmann, The Machine Learning &Global Health network, Swapnil Mishra, Samir Bhatt, Seth Flaxman, Elizaveta Semenova, Xenia Miscouridou, Juliette Unwin, Dino Sejdinovic, Nuno Faria, David A. Duchene, Moritz Kraemer, Sebastian Vollmer

**Affiliations:** 1 Department of Mathematics, Imperial College London, London, UK; 2 National University of Singapore, Saw Swee Hock School of Public Health, Singapore, Singapore; 3 University of Münster, Institute of Epidemiology and Social Medicine, Münster, Germany; 4 National University of Singapore, Institute of Data Science, Singapore, Singapore; 5 Department of Infectious Disease Epidemiology, Imperial College London, London, UK; 6 School of Public Health, University of Copenhagen, Copenhagen, Denmark; 7 Department of Computer Science, University of Oxford, Oxford, UK; 8 Department of Epidemiology and Biostatistics, Imperial College London, London, UK; 9 Department of Mathematics and Statistics, University of Cyprus, Nicosia, Cyprus; 10 School of Mathematics, University of Bristol, Bristol, UK; 11 School of Computer and Mathematical Sciences, The University of Adelaide, Adelaide, Australia; 12 MRC Centre for Global Infectious Disease Analysis, Imperial College London, London, UK; 13 Department of Public Health, University of Copenhagen, Copenhagen, Denmark; 14 Department of Biology, Univesity of Oxford, Oxford, UK; 15 Technical University of Kaiserslautern, Kaiserslautern, Germany; 16 German Research Center for Artificial Intelligence, Kaiserslautern, Germany; 17 Department of Statistics, University of Warwick, Coventry, UK

**Keywords:** Statistical methods, Influenza virus, Viral infection

## Abstract

Social contact surveys are an important tool to assess infection risks within populations, and the effect of non-pharmaceutical interventions on social behaviour during disease outbreaks, epidemics, and pandemics. Numerous longitudinal social contact surveys were conducted during the COVID-19 era, however data analysis is plagued by survey fatigue, a phenomenon whereby the average number of social contacts reported declines with the number of repeat participations and as participants’ engagement decreases over time. Using data from the German COVIMOD Study between April 2020 to December 2021, we demonstrate that survey fatigue varied considerably by sociodemographic factors and was consistently strongest among parents reporting children contacts (parental proxy reporting), students, middle-aged individuals, those in full-time employment and those self-employed. We find further that, when using data from first-time participants as gold standard, statistical models incorporating a simple logistic function to control for survey fatigue were associated with substantially improved estimation accuracy relative to models with no survey fatigue adjustments, and that no cap on the number of repeat participations was required. These results indicate that existing longitudinal contact survey data can be meaningfully interpreted under an easy-to-implement statistical approach addressing survey fatigue confounding, and that longitudinal designs including repeat participants are a viable option for future social contact survey designs.

## Introduction

Despite advances in medicine and public health, infectious respiratory diseases such as coronaviruses, diphtheria, influenza, measles or tuberculosis continue to cause substantial global morbidity^1^ and mortality^2^. These pathogens are easily transmitted via close-range human-to-human contacts, which may be direct via physical contact or indirect through airborne droplets and aerosols^3,4^, and for this reason have high pandemic potential. Consequently, it is fundamental for pandemic preparedness to understand the structure and intensity of human social contacts – across the full diversity of human populations globally^5^. Contact data are used to provide real-time estimates of key quantities such as epidemic reproduction numbers^6^, to optimise vaccine allocation^7^, to estimate transmission risk factors^8^, to provide nuanced assessments into the population groups that drive transmission^9–11^, and identifying efficacious strategies to curb disease transmission^6,12–15^.

Protocols for collecting social contact data through surveys are open-source^16^, and have been systematically implemented internationally during the COVID-19 pandemic^17^. There are several data collection features that obscure the true structure and intensities of contacts, most notably that participants are reporting the age of their contacts in coarse age brackets; that contacts of individuals under the age of 18 years are frequently reported by caregivers; that the demographic composition of the survey panels is updated over time (although individual-level survey weights are available); and that repeat participants who contribute to consecutive surveys tend to under-report their contacts, a phenomenon termed survey fatigue^17,18^. Previous studies have tackled the first two issues. Dan et al.^19^ demonstrated it is possible to estimate contact intensities by 1-year age bands from coarse data given reciprocity constraints, and also that it is possible to leverage data from adults to decipher the contacts of children. Post-stratification methods can also be used further to incorporate survey weights^6,20^. However, it remains unclear to what extent it is possible to adjust for the confounding effects of survey fatigue after the data have been collected^19,21–23^, and consequently how future longitudinal contact surveys should best be implemented to avoid or mitigate such effects.

Here, we investigate contact data collected longitudinally over 33 survey waves from April 2020 to December 2021 in Germany through the COVIMOD Study to address the following questions: What are the individual determinants of survey fatigue; is it possible to control for individual-level survey fatigue in statistical models aimed at estimating the structure and intensity of social contacts; and is there an upper limit on repeat participation beyond which the error introduced through survey fatigue becomes challenging to control for in statistical models. The COVIMOD surveys were conducted using the similar protocol as all countries participating in the European CoMix Study. For this reason, we expect our investigations will generally improve our ability to interpret social contact data already collected, our ability to design future longitudinal contact surveys that are robust to survey fatigue effects, and accuracy in infectious disease incidence forecasting^24^.

## Results

**Figure 1:**
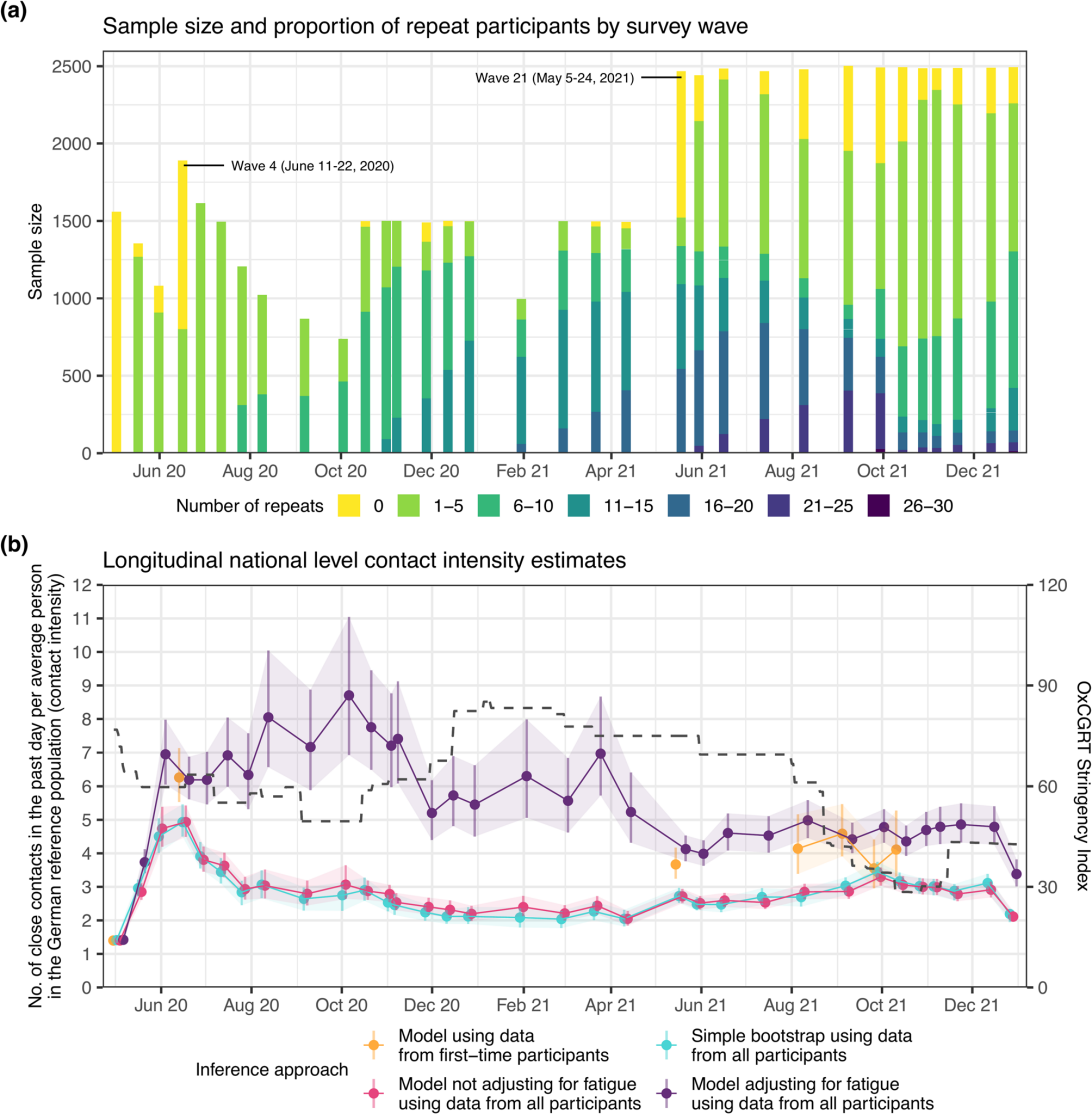
Longitudinal contact intensity estimates during the COVID-19 pandemic in Germany. (**a**) Longitudinal structure of 33 survey waves of the COVIMOD social contact study, showing sample sizes for each survey wave on the y-axis. Colours indicate the number of repeat participations. (**b**): Longitudinal, national-level contact intensity estimates (point: simple bootstrap mean or posterior median estimate, linerange: 95% bootstrap confidence or 95% bayesian credible intervals) are shown according to different estimation approaches: Bayesian model using data from first-time participants only, for waves with more than 300 first-time participants (orange); simple bootstrap using data from all participants and not accounting for survey fatigue (blue), Bayesian model using data from all participants and not adjusting for survey fatigue (pink); Bayesian model using data from all participants and adjusting for survey fatigue (purple). The dashed line represents the OxCGRT Stringency Index with higher values indicating a higher degree of contact restrictions (min: 0, max: 100).

### Longitudinal and subgroup-specific contact intensity estimates during the pandemic

In this section, we highlight the main findings of the study: the impact of survey fatigue on estimates of social contact intensity during the COVID-19 pandemic. We intentionally abstract away technical details and preceding analyses, which are presented in subsequent results and methods sections.

Between April 30, 2020, and December 31, 2021, the COVIMOD study collected self-reported close contact data from 7,851 participants, totaling 141,928 contacts. Each participant reported the number of people they had close contact with on the previous day. A close contact is defined as either a skin-to-skin contact or the exchange of three or more words in the presence of another person. We define *first-time participants* as individuals completing the survey for the first time in a given wave, and *repeat participants* as those who had previously participated in one or more waves.

Figure 1a shows the timeline of survey waves, the number of participants per wave, and the proportion who had participated before. Figure 1b displays estimates of average population-level contact intensity–defined as the average number of close contacts per person per day–along with 95% bayesian credible intervals. These estimates were obtained using four approaches: (i) a model fitted to data from first-time participants only; (ii) a simple parametric bootstrap on data from first-time participants ^25^; (iii) a model using data all participants but not adjusting for survey fatigue; and (iv) a model using data from all participants with adjustments for survey fatigue. Importantly, first-time participants are not subject to survey fatigue, making them a key reference group. Comparing model-based estimates with those derived from first-time participant data is essential for evaluating the effectiveness of fatigue correction.

**Figure 2:**
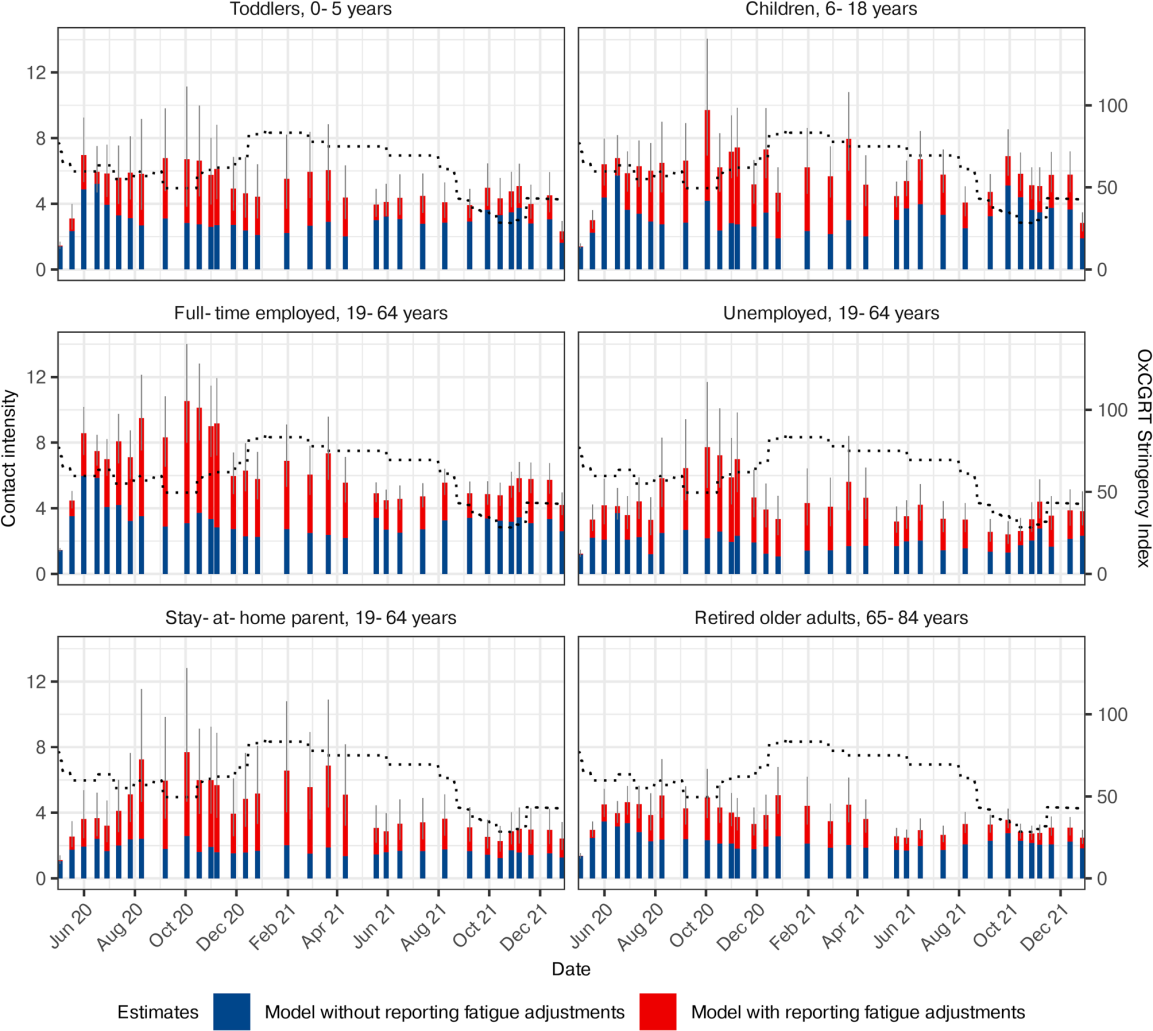
Comparison of subgroup specific contact intensity estimates with and without adjustments for survey fatigue. Posterior median contact intensity estimates for eight population subgroups, comparing the fatigue-adjusted model (red) to the unadjusted model (blue). The 95% credible intervals of the adjusted estimates are shown as lineranges. Dotted lines denote the OxCGRT stringency index ^26^. Supplemental Figure 2 presents the percent relative corrections from the adjusted model relative to the unadjusted model.

**Figure 3:**
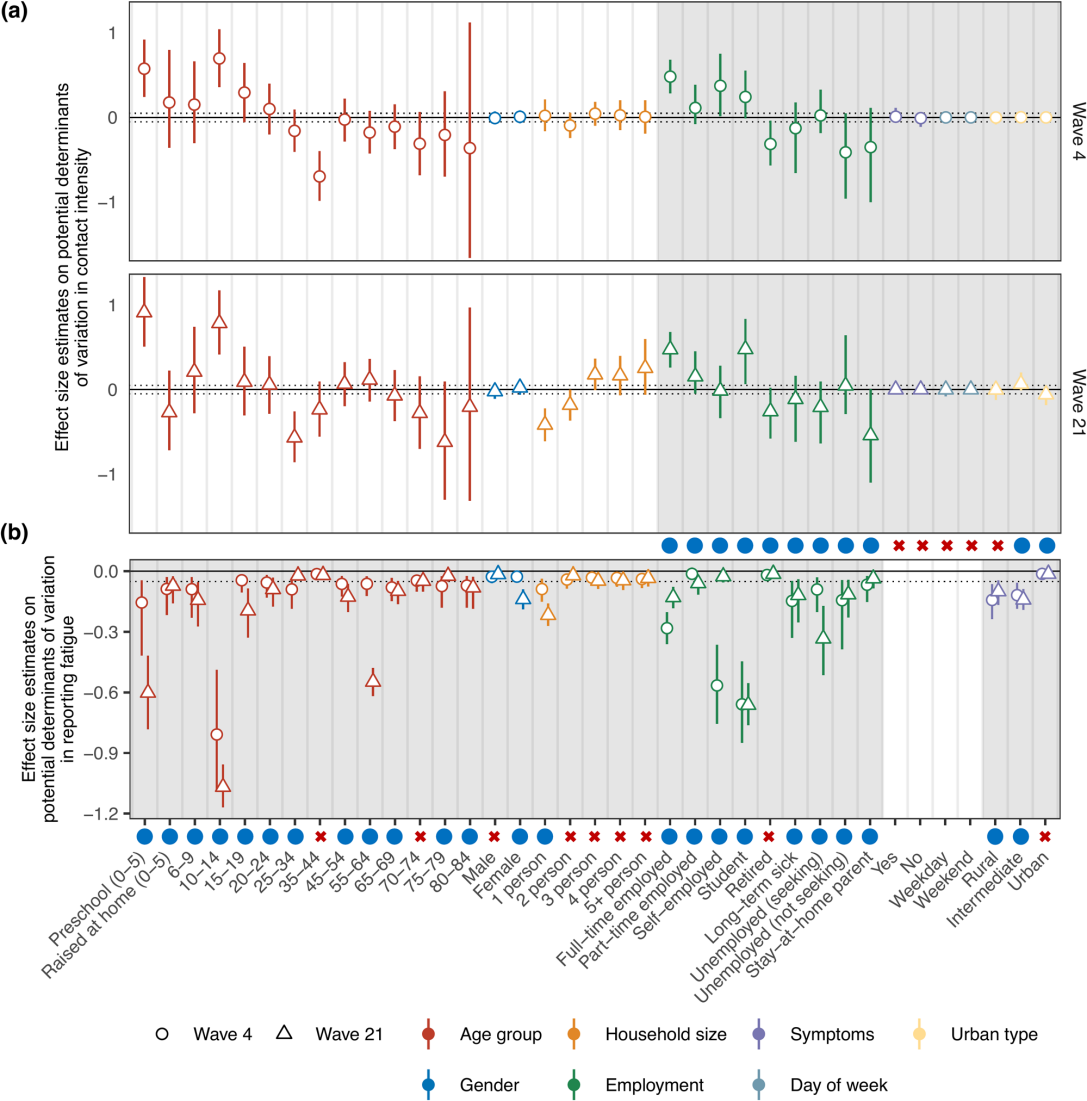
Effect size estimates on determinants of variation in contact intensity and survey fatigue in the feature selection models. (**a**) Posterior effect size estimates on contact intensity determinants in the feature selection model on survey data from survey waves 4 and 21 (circles and triangles: posterior median estimate in the Bayesian model using data from first-time participants, line-range: corresponding 95% credible intervals) relative to the overall baseline contact intensity term in the model. Gray solid lines denote the average contact intensity, and gray dotted lines the feature selection cut-off threshold ($>\pm 5\%$ change). White background indicates features that were always included in the model, and grey background indicates features that were tested for inclusion through Bayesian feature selection. Blue solid dots denote selected variables and red crosses denote variables that were not identified to have $>\pm 5\%$ deviations in average contact intensities. (**b**) Posterior effect size estimates on survey fatigue determinants in the feature selection model on survey data from survey waves 4 and 21 (points: posterior median estimate in the Bayesian model using data from repeat participants, line-range: corresponding 95% credible intervals) relative to no effect. Other plot features are as in sub-figure (**a**), except that the gray solid lines denotes no change in contact intensity as compared to first-time participants in the same subgroup, and the gray dotted line denotes the feature selection cut-off threshold ($> 5\%$ decrease).

In most survey waves, the fatigue-adjusted estimates (purple) closely matched those obtained from first-time participants using the same model (orange) and from the bootstrap method (Supplementary Figure S1). In contrast, estimates from the same model applied to all participants without fatigue adjustments (pink), as well as those derived using the bootstrap method, showed substantial under-reporting in contact intensity. To investigate subgroup effects, we compared estimates from the fatigue-adjusted and unadjusted models across specific populations (Fig. 2). We hypothesized that under-reporting due to fatigue would disproportionately affect groups such as preschool children (0–5 years), school-aged children (6–18 years), students, and self-employed adults (Fig. 3b). This was confirmed by the fatigue-adjusted model, which provided substantial corrections to longitudinal contact intensity estimates, reflecting the complex participation structure of the cohort (Fig. 2, Supplementary Figure S2).

### Determinants of survey fatigue and pandemic contact intensities

In this and the subsequent subsections, we present the results of the analyses that underpin our primary findings. First, we identify individual-level predictors of contact intensity and survey fatigue. Next, we determine the appropriate functional form for modelling survey fatigue. Finally, we evaluate the accuracy of our proposed correction technique.

Two survey waves, wave 4 and wave 21, included substantial numbers of both first-time and repeat participants (Fig. 1a). These waves allowed us to identify individual-level features associated with variations in contact intensity during the pandemic. We then examined how survey fatigue is associated with these features among repeat participants surveyed at the same time point. Specifically, wave 4 (June 11 to June 22, 2020) included 1,089 first-time participants and 801 repeat participants, and wave 21 (May 5 to May 24, 2021) included 947 first-time and 1,521 repeat participants. We performed a Bayesian feature selection analysis with sparsity inducing priors ^27,28^ to identify features associated with changes in pandemic contact intensities, and features associated with a reduction in contact intensities in connection with repeat participation. We first analysed participants from wave 4, and then repeated the analysis independently on data from wave 21 to test the robustness in the features selected across different phases of the pandemic. Well-known determinants of contact intensities are age ^6,14,17,19,21,29^, sex ^14,17,19^, and household size ^6,14,17,21^, and we always included these features as controls in our models. Potential further determinants that were previously investigated and also measured during the COVIMOD study included employment status ^6,17^, presence of disease symptoms, day of week effects ^14^, and urban-rural typology (rural, intermediate, or urban), and these factors were tested for association with variation in contact intensities.

**Table 1:** Determinants of social contact intensities and survey fatigue in Germany, June 11-22 2020 (wave 4) and May 5-24 2021 (wave 21).

		# Participants (# repeat)^*1^				
Variable	Category	Survey Wave 4	Survey Wave 21	Well- $\text {known}^{*2}$	Relevant to contact $\text {intensity}^{*3}$	Relevant to reporting $\text {fatigue}^{*4}$
Age group	Preschool (0-5)	61 (8)	69 (33)	$\circ$	*n.t.*	$\circ$
	Raised at home (0–5)	17 (2)	44 (15)	$\circ$	*n.t.*	$\circ$
	6-9	34 (8)	51 (28)	$\circ$	*n.t.*	$\circ$
	10-14	80 (25)	112 (71)	$\circ$	*n.t.*	$\circ$
	15-19	103 (27)	145 (72)	$\circ$	*n.t.*	$\circ$
	20-24	106 (16)	151 (81)	$\circ$	*n.t.*	$\circ$
	25-34	216 (54)	277 (172)	$\circ$	*n.t.*	$\circ$
	35-44	163 (68)	233 (157)	$\circ$	*n.t.*	$\times$
	45-54	271 (139)	353 (209)	$\circ$	*n.t.*	$\circ$
	55-64	318 (197)	395 (250)	$\circ$	*n.t.*	$\circ$
	65-69	311 (161)	382 (255)	$\circ$	*n.t.*	$\circ$
	70-74	118 (61)	174 (128)	$\circ$	*n.t.*	$\times$
	75-79	48 (24)	52 (34)	$\circ$	*n.t.*	$\circ$
	80-84	5 (3)	9 (5)	$\circ$	*n.t.*	$\circ$
Sex	Male	965 (391)	1288 (807)	$\circ$	*n.t.*	$\times$
	Female	886 (402)	1159 (703)	$\circ$	*n.t.*	$\circ$
Household size	1 person	477 (334)	728 (457)	$\circ$	*n.t.*	$\circ$
	2 person	435 (165)	825 (465)	$\circ$	*n.t.*	$\times$
	3 person	535 (199)	497 (339)	$\circ$	*n.t.*	$\times$
	4 person	206 (53)	276 (167)	$\circ$	*n.t.*	$\times$
	5+ person	198 (42)	121 (82)	$\circ$	*n.t.*	$\times$
Employment	Full-time employed	508 (220)	702 (423)		$\circ$	$\circ$
	Part-time employed	202 (96)	261 (166)		$\circ$	$\circ$
	Self-employed	89 (48)	106 (69)		$\circ$	$\circ$
	Student (15+)	193 (45)	228 (130)		$\circ$	$\circ$
	Retired	462 (248)	615 (422)		$\circ$	$\times$
	Long-term sick	42 (21)	59 (33)		$\circ$	$\circ$
	Unemployed (seeking)	82 (32)	91 (49)		$\circ$	$\circ$
	Unemployed (not seeking)	34 (10)	50 (37)		$\circ$	$\circ$
	Stay-at-home parent	47 (30)	59 (34)		$\circ$	$\circ$
COVID-like Symptoms	Yes	468 (182)	620 (315)		$\times$	*n.t.*
	No	1383 (611)	1827 (1195)		$\times$	*n.t.*
Day of week	Weekday	1579 (569)	2359 (1433)		$\times$	*n.t.*
	Weekend	272 (224)	88 (77)		$\times$	*n.t.*
Urban type	Rural	282 (119)	376 (232)		$\times$	$\circ$
	Intermediate	705 (291)	923 (564)		$\circ$	$\circ$
	Urban	864 (383)	1148 (714)		$\circ$	$\times$
	**Total**	1851 (793)	2447 (1510)			
*n.t.*: Not tested, $\circ$: Selected, $\times$: Not selected						
^*1^ The total number of participants is reported on the left and the number of repeat participants is reported in brackets.						
^*2^ By “well-known” we refer to covariates which have been extensively documented in previous studies.						
^*3^ Relevant features are those which increase or reduce contact intensity by more than 5% relative to the baseline.						
^*4^ Relevant features are those which reduce contact intensity by more than 5% relative to the baseline.						

Table 1 lists all features that we investigated, the total sample sizes for each respective subgroup in the two waves for first-time and repeat participants, the number of repeat participants (in brackets), and the identified determinants of contact intensities and survey fatigue. In our analyses, we stratified children aged 0-5 years by whether they were raised at home or attended pre-school due to the well-established influence of school attendance on child contact intensities ^30^. Households of size 5 or more were grouped together, and comprised 10.7% and 4.9% of all households in the sample for survey wave 4 and wave 21, respectively. In addition to age, sex, and households size, we found from the survey data of first-time participants that working (full-time, part-time, or self-employed), being a student, and living in intermediate or urban areas were associated with a sample-level increase in average contact intensities by more than 5% (Table 1, Fig. 3a). Conversely, being a stay-at-home parent, unemployed, retired, suffering from long-term sickness or being disabled were associated with decreased average contact intensities of more than 5%. The features that were associated with >5% deviations from average contact intensities were qualitatively similar between the two waves (Table 1, Fig. 3a).

Next, we quantified the impact of survey fatigue on contact intensities, defined as a reduction in contacts among individuals who had participated in previous survey waves compared to first-time participants within the same population subgroup. We extended our Bayesian model to include survey fatigue terms for age, sex, household size, employment status, and urban-rural typology. Feature selection priors ^27^ were applied to these terms, and the model was fitted to data from both first-time and repeat participants in wave 4, and subsequently in wave 21 to assess robustness (see Methods). We retained features associated with a posterior median reduction of more than 5% in contact intensities among repeat participants relative to first-time participants in either wave 4 or wave 21. Table 1 lists the selected features, and Fig. 3b shows the estimated reduction in contact intensity for repeat participants. Among age groups, only those aged 35-44 and 70-74 were *not* associated with a reduction in average contacts. Women and individuals living alone (household size 1) showed reduced contact intensity. For employment status, all categories except “retired” were associated with lower contact intensity. Living in rural or intermediate areas was also linked to reduced contacts. The set of features associated with $>5\%$ reductions differed only slightly between waves 4 and 21.

**Figure 4:**
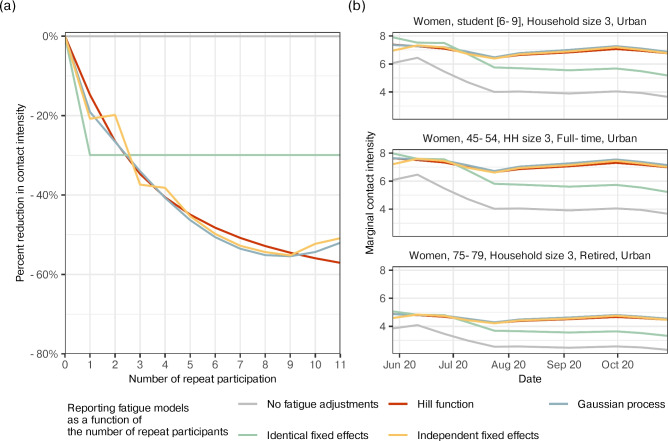
Dynamics in the severity of survey fatigue with increasing repeat participation in the COVIMOD Study. (**a**) Estimated percent reduction in contact intensity as a function of the number of repeat participation for no survey fatigue adjustments (gray), the Hill model (red), the Gaussian process model (blue), the identical fixed effects model (green), and the independent effects model (yellow) (line: posterior median estimate). (**b**) Estimated longitudinal contact intensities for women aged 6-9, 45-54, and 75-79 years living in an urban 3-person household for no survey fatigue adjustments (gray), the Hill model (red), the Gaussian process model (blue), the identical fixed effects model (green), and the independent effects model (yellow).

### Impact of the number of repeat participations on survey fatigue severity

We examined how survey fatigue varied with the number of participations using data from waves 3–12 (June to October 2020). Among participants, $n=1{,}287$ were first-time respondents, $n=8,057$ had participated 1–5 times, $n=3,397$ had participated 6–10 times, and $n=90$ had participated 11 times. Figure 4a shows in yellow, the estimated percent reduction in contact intensity from a longitudinal model that includes separate fixed effect terms for each repeat participation count $r = 0, 1, \dotsc , 11$. This model allows for distinct survey fatigue effects at each level of repeat participation. The estimates reveal a clear pattern: under-reporting due to survey fatigue worsens with increasing participation.

To develop a parsimonious model of survey fatigue dynamics, we assessed three modelling approaches: a identical fixed effects model ^21^, a non-parametric Gaussian process (GP) model, and a parametric Hill model frequently used in bio-chemistry applications ^31^. While the identical fixed effects model over-simplified survey fatigue dynamics, the remaining two approaches gave similar results (Fig. 4a) that also match results from the independent fixed effects model, indicating that fatigue dynamics can be well-approximated with Hill functions and it is not necessary to resort to more computationally intensive non-parametric techniques.

The posterior median and 95% CI for the parameters of the Hill function were $\gamma$: 1.18 [0.83, 1.93], $\zeta$: -1.90 [-2.44, -1.43], and $\eta$: 1.16 [0.81, 1.73], indicating that the maximum percentage reduction in the number of reported contacts due to repeat participation is approximately $100 \times (e^{-\gamma } - 1) = -69.5\%\ [-85.5\%, -56.3\%]$. Figure 4b illustrates the impact of adjusting for survey fatigue on longitudinal contact intensity estimates in three population subgroups, women of age 6-9, 45-54, and 75-79 years living in an urban 3-person household. With the exception of the identical fixed effects model, all models with survey fatigue adjustments suggest that contact intensities remained steady through waves 3 to 12 (June to October, 2020), in contrast to unadjusted estimates that exhibit notable decreases as the proportion of repeat participants in the sample increase (Fig. 1).

**Figure 5:**
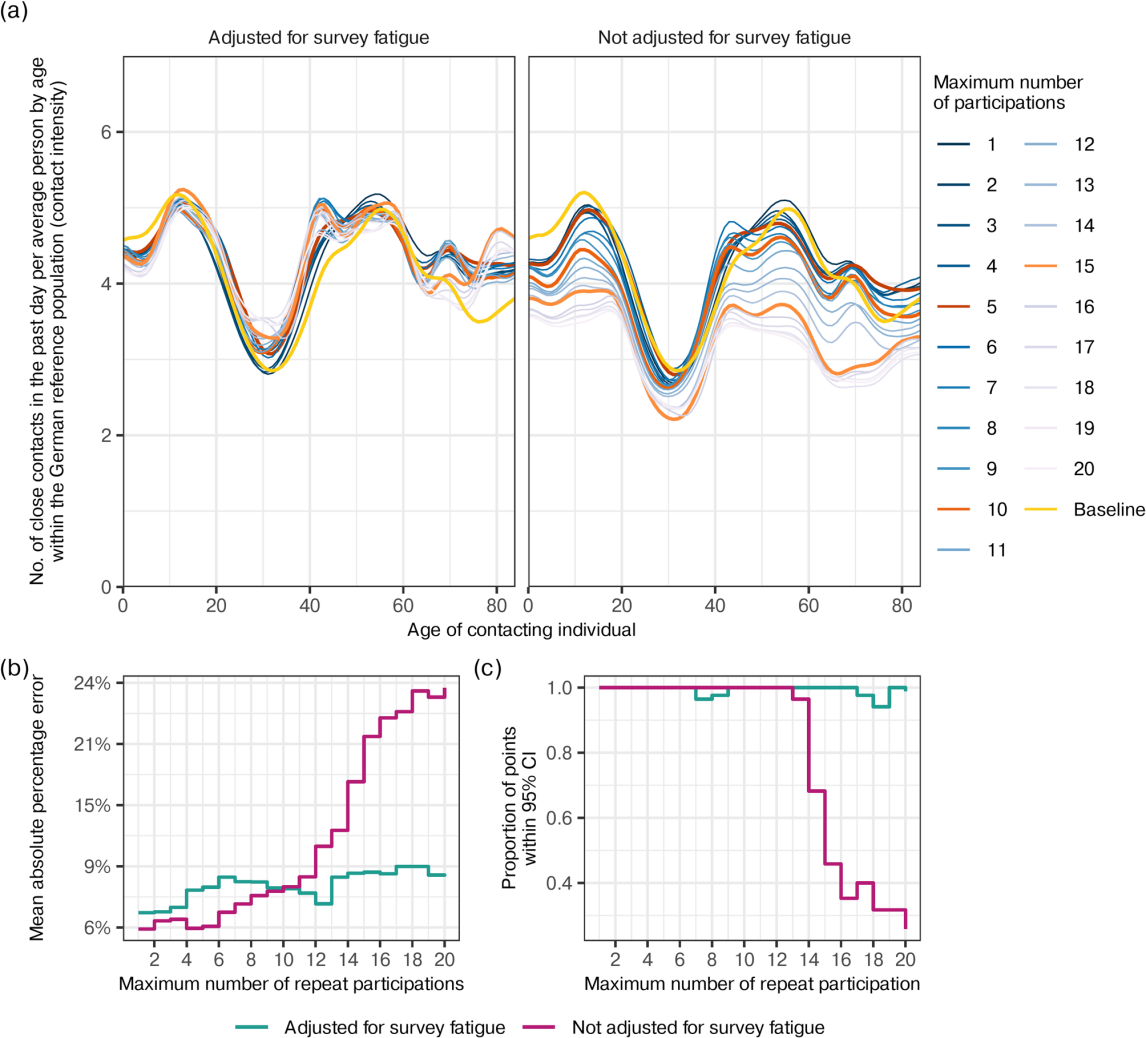
Accuracy of the survey fatigue adjusting model in correcting age-specific contact intensity estimates from wave 21 of the COVIMOD Study. (**a**) Posterior median contact intensity estimates for ages 0 to 84 during the COVIMOD survey wave 21, obtained from the survey fatigue adjusting model that adjusts for population subgroup-specific survey fatigue effects via separate Hill functions (left) and the same model without survey fatigue adjustments (right). In both panels, the gold curve represent posterior median estimates obtained from first-time participants. Coloured curves represent the posterior median estimates obtained from including participants with increasingly larger maximum number of repeats participations. (**b**) Mean absolute percentage error between the baseline (gold curve) and estimates obtained from the survey fatigue adjusting model on data including participants with more repeats (turquoise) and from the same model without survey fatigue adjustments (purple). (**c**) Proportion of posterior median age-specific contact intensity estimates from data of first-time participants that fell within the 95% credible intervals of the posterior age-specific estimates of the survey fatigue adjusting model on data including participants with more repeats.

### Accuracy in correcting contact intensity estimates from survey fatigue effects

Given the substantial detrimental impacts of survey fatigue on longitudinal contact intensity estimates, we next assessed the possibility of correcting contact intensity estimates by utilising the Hill function approach from the previous section. In the experiment, we aimed at correcting age-specific contact intensities as non-pharmaceutical interventions often target specific age groups ^30^, and compared the accuracy in age-specific contact intensities inferred from data from participants with increasingly many repeat participations to the age-specific contact intensities inferred from first-time participants. We used data from survey wave 21 for which the sample consists of a large proportion of first-time participants and participants with many repeats. We extended our Bayesian model to specify age-specific contact intensities with a Gaussian process on the age of survey participant, and assigned independent Hill functions to every fatigue feature selected in the previous analysis (Methods).

The model was initially applied to data with first-time participants only to serve as a baseline which excludes the confounding effects of survey fatigue. Individuals who participated in previous survey rounds were then incrementally added back into the sample to understand the ability of the survey fatigue adjusting model to correct contact intensity estimates. Figure 5a compares the posterior median age-specific contact intensity estimates from first-time participants (gold) to those obtained by incrementally including groups of participants with more repeats, in both a model that adjusts for survey fatigue (left) and a model that does not adjust for survey fatigue (right).

The mean absolute percentage error (MAPE) between the contact intensity estimates from first-time participants and those from data including repeat participants is shown in Fig. 5b, and reveals a marked difference in estimates from the adjusting and non-adjusting models. The MAPE from the non-adjusting model increased steadily with the maximum number of repeat participations and reaches approximately 24% with the inclusion of all participants, whereas estimates from the adjusting model consistently had a MAPE around 7%-9%. Figure 5c examines the proportion of the age-specific contact intensity estimates from first-time participants that fell within the 95% posterior credible intervals of the age-specific estimates obtained from data including participants with increasingly many repeat participations. In the models without adjustment for survey fatigue, coverage decreased noticeably after incorporating data from participants with 15 repeats. In contrast, the adjusting model maintained $\approx$100% coverage regardless of the maximum number of repeat participations. Past these global assessment measures, Fig. 5a indicates estimates were most sensitive to survey fatigue in age ranges 0-20 years and 40-70 years which are highly relevant in disease transmission ^30^. In models without survey fatigue adjustments, capping data to individuals with up to 5, 10, and 15 repeat participations resulted in a MAPE of 5.3%, 8.9%, 22.7% leaving out 52.3%, 16.8%, and 5.8% of responses, respectively. In contrast, in the model with survey fatigue adjustments, capping at the same thresholds resulted in a MAPE of 5.4%, 6.8%, and 5.8% in these age groups.

## Discussion

In this work, we tackled three pivotal questions aimed at enhancing our ability to understand temporal changes in social contacts in a pandemic from longitudinally collected surveys: First, what are the primary individual characteristics associated with survey fatigue; second, is it possible to correct contact intensity estimates from under-reporting effects due to survey fatigue; and third, what is the impact on contact estimates throughout the COVID-19 pandemic when we adjust for survey fatigue. Our findings indicate that survey fatigue is particularly relevant in children, middle age individuals, and the working population (Figs. 3, 5). We identify the functional form of survey fatigue and introduce the use of Hill functions as a viable modelling method to mitigate its detrimental effects (Fig. 4). We show that the effects of survey fatigue differs across different population subgroups and also changes dynamically over time (Fig. 2). Our model’s adjustments for survey fatigue suggest that social contact intensities were higher than those estimated by non-adjusting methods, and that these adjustments bring estimates closer to those estimated using data from first-time participants alone (Figs. 1, 5).

Recruiting participants multiple times in longitudinal web-based social contact surveys is necessary as opposed to enlisting a new panel for each wave. In particular, the limited number of members within any given online panel necessitates repeated sampling, as it is only a matter of time before all eligible and willing participants have been recruited. This limitation is particularly challenging when striving for a sample that is approximately representative of the population. While panels from various market research companies could theoretically be employed to circumvent this issue, in practice, it requires each company to independently recruit participants and administer surveys within their panels, leading to substantial organisational, financial, and temporal costs. Allowing participants to be enrolled multiple times not only mitigates these costs but also facilitates the attainment of a sample that closely mirrors the population age and sex structure. Moreover, repeated sampling enables the examination of intra-personal variability in contact behavior over time, an area largely unexplored in pre-pandemic studies ^32^. Despite these benefits, allowing for multiple participation presents significant challenges in the form of survey fatigue. Multiple studies across different countries have shown that on average, the relative number of contacts reported by individuals decrease with the number of participations in the survey ^17,19,21^. This limits our ability to accurately understand contact dynamics which in turn limits our ability to accurately assess infection risk and estimate reproduction numbers ^33–35^, which is crucial for targeted non-pharmaceutical interventions, vaccinations strategies, and preparations for future pandemics.

Our results suggest that parents reporting children contacts (parental proxy reporting), students (aged 19+), middle-aged individuals, those in full-time employment and those self-employed were the subgroups most effected by survey fatigue. We postulate that the relative reduction in reported contacts among children is primarily due to the survey fatigue experienced by parents, who report on behalf of their children. Moreover, parents face notable challenges in reporting their children’s social interaction at school and other educational settings, where direct parental supervision is uncommon. This significantly hinders parents’ ability to accurately account for all of their children’s contacts, even under the best circumstances. Students and middle-aged working individuals may be the subgroups most affected by recall bias as they may find it hard and/or tiresome to recall and report a large number of contacts at school/work place and other non-household settings. Designers of future social contact studies may benefit from these observations and take informed design decisions to mitigate bias in these specific subgroups.

For large-scale pandemic-era social contact studies, which must be conducted online for efficiency in scale and timing, implementing thorough quality control may not always be feasible, and no amount of meticulous design may prove sufficient to eliminate survey fatigue bias. For data that has already been collected, modelling is necessary to correct for the biases discussed above. Our longitudinal models applied data from wave 3 to 12 (Fig. 4) and empirical results from past studies ^17^ suggests that fatigue effects should be modelled as a monotonically decreasing non-linear function of the number of repeat participation. While numerous candidates exist, we propose using the Hill function for its intuitive interpretation as a dose-response model, where administering the survey is considered a dose and fatigue is the response ^31^. Despite imposing strong assumptions on the functional form of fatigue effects, the Hill function offers computational advantages over non-parametric alternatives like Gaussian processes, as it requires estimating only three parameters. An application of the Hill function approach on data from wave 21 shows that it can effectively mitigate the effects of survey fatigue. This allows us to make use of a larger portion of the collected data with less fear of obtaining significantly under-estimated values.

The findings of this study should be considered in the context of several limitations. With regards to the collected data, despite quota sampling, the final COVIMOD samples were not fully representative of the German population. Middle-aged individuals with children were under-represented, as many surveys were completed by parents on behalf of their children. Moreover, COVIMOD was conducted as an online survey, with participants recruited from an online market research panel through email, which may introduce bias as those with internet access who take part in health-related surveys might exhibit a higher compliance with social distancing measures. The retrospective nature of the reporting may also reduce the accuracy of reports as participants face challenges in recalling past events. Further details on other potential sources of bias in COVIMOD and strategies employed to address them is available elsewhere^14^.

Second, with regards to our methodological approach, accuracy estimates were obtained based on estimates obtained with data from first-time participants. However, these participants may not always be representative of the sample or population. Despite this limitation, we argue that using first-time participants’ data is the most viable option in the absence of a definitive method to establish the absolute truth. Additionally, we applied Hill function-based adjustments for each variable identified during the feature selection stage, allowing us to explore differences in survey fatigue across various population subgroups (Fig. 2). However, as indicated in Fig. 1, the variables associated with survey fatigue may vary across waves and are additionally potentially heterogeneous across individuals in terms of non-measured factors. Future research is needed to investigate the extent of individual-level survey fatigue effects beyond the co-variates that we considered.

Finally, the observed reduction in reported contacts may not be solely attributable to survey fatigue, but could also reflect differences between individuals who remain in the study and those who drop out. As we lack data on response rates, a formal non-response analysis was not feasible. However, previous work has examined dropout patterns in a closely related longitudinal social contact study conducted in Belgium during the COVID-19 pandemic ^21^. Similarly, a separate study in Germany from the same period suggests that individuals with fewer social contacts are more likely to drop out ^36^.

Future work should explore additional longitudinal surveys, such as CoMix^6,37^. Alternative statistical approaches may also be valuable. For example, treating first-time and repeat participants as separate datasets and applying modular or semi-modular inference ^38^ could reduce contamination from repeat respondents, though it requires careful calibration of information sharing. In waves with few repeats, modelling fatigue with independent fixed effects remains practical. However, based on our findings, we advise against arbitrarily capping repeats (e.g., 5 or 10) and estimating contact intensity without model-based adjustments; both previous studies ^19,21^ and our analyses (Fig. 1, Figure S1) show that including participants with even a small number of repeats may lead to substantial underestimating of contact intensities.

In conclusion, this study identifies key individual-level features influencing overall contact intensity, and those associated with survey fatigue. We find simple-to-implement Hill functions can accurately control for survey fatigue effects that occur in contemporary longitudinal social contact survey designs. While this methodology may not completely eliminate the bias induced by survey fatigue, it enables us to use the entire dataset with reduced concern about underestimating contact intensity. Our findings highlight that existing longitudinal contact survey data can be meaningfully interpreted despite survey fatigue effects, and that longitudinal designs including repeat participants are a viable option for future social contact survey designs. By refining the accuracy of contact estimates, model-based statistical approaches can better inform public health strategies and interventions at high resolution beyond age and gender, and thereby contribute to more effective management and mitigation of contact-related infectious disease outbreaks in the future.

## Methods

### The COVIMOD study

The COVIMOD study is a repeated survey from April 2020 to December 2021 comprising 33 waves. Participants were recruited through email invitations sent to existing panel members of the online market research platform IPSOS i-Say^39^. To ensure the sample’s broad representativeness of the German population, quota sampling was conducted by age, sex, and region. A subset of adult participants living with children under the age of 18 were selected to answer the survey on behalf of their children. Similar to the CoMix study^6,13^, individuals were invited to participate in multiple waves to track changes in behaviour and attitude toward COVID-19 across time. When the participant size did not meet the sampling quota due to dropout, new participants were recruited into the study.

The COVIMOD questionnaire was based on the questionnaire of the CoMix study^6,13^, and includes questions on demographics, the presence of a household member belonging to a high-risk group, attitudes towards COVID-19 as well as related government measures ^14,37^. Participants were asked to provide information about their social contacts between 5 a.m. the previous day to 5 a.m. the day of answering the survey. A contact was defined as either a skin-to-skin contact (e.g., kiss, handshake, hug), or the exchange of three or more words in the presence of another person. Participants reported information on the sex and age band of each contact, their relationship, the contact setting (e.g., home, school, workplace, place of entertainment), and whether the contact was a household member.

For survey waves 1 and 2, participants were asked to report information on each contact separately. However, from wave 3 participants were provided with the option to report the total number of contacts in the event that they could not list all of them separately. A copy of the COVIMOD questionnaire may be found in Additional file 1 of Tomori et al. 2021^14^. COVIMOD was approved by the ethics committee of the Medical Board Westfalen-Lippe and the University of Münster, reference number 2020-473-fs. Written informed consent was obtained from all participants and/or their legal guardians. All methods were carried out in accordance to relevant guidelines and regulations. As only anonymised COVIMOD data was used in this work, an institutional review was not required for reanalysis.

### Other datasets

To better contextualise the changes in social contact patterns in relation to non-pharmaceutical interventions, we acquired data from the Oxford Covid-19 Government Response Tracker (OxCGRT) which collected information on policy measures that tackle COVID-19 through 2020 to 2021 ^26^. Specifically, we extract the OxCGRT compact dataset for Germany and use the stringency index which measures containment, closure policies, and public information campaigns. The urban-rural typologies of where participants lived were determined by matching residence information to nomenclature of territorial units for statistics (NUTS) level 3 regions and using the classification defined by Eurostat. Specifically, predominantly urban regions are where at least 80% of the population live in urban clusters (a urban-rural typology of at least 300 inhabitants per $\text {km}^2$ and a minimum population of at least 5000 inhabitants); intermediate regions are defined as regions where between 50% but to 80% of the population live in urban clusters; and predominantly rural regions are defined as regions where least 50% of the population live in rural areas (all areas outside urban clusters) ^40^. NUTS data for 2021 were downloaded from the Eurostat website ^41^. To construct post-stratification weights we obtained population size estimates by age and sex for 2019 (Table code: 12411-0041) and household size estimates for 2019 (Table code: 12211-9020) from the GENESIS-online database ^42^, and population size by NUTS level 3 region for 2019 (Table code: demo_r_pjangrp3) from Eurostat ^43^.

### Software

All analysis in this work was performed using R version 4.4.1 ^44^. Bayesian inference was performed using the *Stan*^45^ probabilistic programming language through the *cmdstanr*^46^ package version 2.34.1 as front-end. A number of analyses were performed on high performance computing clusters maintained by the research computing service at Imperial College to reduce experiment time but all analysis can be run on modern laptops. The data and code to replicate the results in this study can be found at: https://github.com/ShozenD/contact-survey-fatigue.

### Data processing

In all analyses, we omitted data for participants who did not disclose their age or sex, information which is required in all of our models. Adhering to ethical standards, the age of participants under 18 years (children) were reported in distinct age groups, namely, 0–4, 5–9, 10–14, and 15–18 years. To ascertain detailed age data for those under 18, we imputed their age by selecting from a discrete uniform distribution framed by the minimum and maximum ages of the participant’s age group. The total number of contacts reported by individuals were truncated to 50 (99th percentile) to mitigate the effects of extreme outliers on statistical estimates.

### Features associated with pandemic social contacts

The number of contacts by an individual within a given time-frame can influenced by a variety of individual-level factors including age ^6,14,17,19,21,29^, sex ^14,17,19^, household size ^6,14,17,21^, employment status ^6,17^, and day of week effects ^14^. We perform a feature selection analysis with a focus on employment status (full-time employed, part-time employed, stay-at-home parent, long-term sick, retired, self-employed, student (above age 15), unemployed and seeking job, unemployed and not seeking job), day of week effects (weekday, weekend), presence of COVID-like symptoms (yes, no), and urban-rural typology (rural, intermediate, urban) while controlling for the effects of age, sex, and household size (Table 1). To prevent survey fatigue effects from contaminating the results, only records from first-time participants were used in the analysis.

Let $Y^{(0)}_i$ for $i = 1,2,\ldots , n^{(0)}$ denote the number of contacts by a first-time participant. We model $Y^{(0)}_i$ using a negative binomial distribution, $Y^{(0)}_i \sim \operatorname {NegBinomial}(\lambda _i, \varphi )$ ($i = 1,2,\ldots ,n^{(0)}$), where $\mathbb {E}[Y^{(0)}_i] = \lambda _i$ and $\operatorname {Var}(Y^{(0)}_i) = \lambda _i + \lambda ^2_i/\varphi$. The mean $\lambda _i$ is modelled on the log scale as

1 $$\begin{aligned} \log (\lambda _i) = \beta _0&+ \underbrace{ {\varvec{u}}_{i,\text {age}}^\top {\varvec{\alpha }}_{\text {age}} + {\varvec{u}}_{i,\text {sex}}^\top {\varvec{\alpha }}_{\text {sex}} + {\varvec{u}}_{i,\text {hhsize}}^\top {\varvec{\alpha }}_{\text {hhsize}} }_{\text {control features}} \nonumber \\&+ \underbrace{ {\varvec{v}}_{i,\text {employ}}^\top {\varvec{\beta }}_{\text {employ}} + {\varvec{v}}_{i,\text {symptom}}^\top {\varvec{\beta }}_{\text {symptom}} + {\varvec{v}}_{i,\text {dow}}^\top {\varvec{\beta }}_{\text {dow}} + {\varvec{v}}_{i,\text {urban}}^\top {\varvec{\beta }}_{\text {urban}} }_{\text {test features}}. \end{aligned}$$

The control features ${\varvec{u}}_{i,\text {age}}, {\varvec{u}}_{i,\text {sex}}, {\varvec{u}}_{i,\text {hhsize}}$ are one-hot encodings of age (14 categories), sex (2 categories), and household size (5 categories), respectively. These are standard predictors in contact behaviour studies and are always included in the model without selection. The test features ${\varvec{v}}_{i,\text {employ}}, {\varvec{v}}_{i,\text {symptom}}, {\varvec{v}}_{i,\text {dow}}, {\varvec{v}}_{i,\text {urban}}$ are one-hot encodings of employment status (9 categories), presence of COVID-like symptoms (2 categories), day of week (2 categories), and urban-rural classification (3 categories), respectively. We assign the following priors:

$$\begin{aligned} \beta _0&\sim \operatorname {Normal}(0, 100), \\ {\varvec{\alpha }}_{\text {age}}&\sim \operatorname {SZMVNormal}(14/13), \\ {\varvec{\alpha }}_{\text {sex}}&\sim \operatorname {SZMVNormal}(2), \\ {\varvec{\alpha }}_{\text {hhsize}}&\sim \operatorname {SZMVNormal}(3/2), \\ {\varvec{\beta }}_{\text {employ}}&\sim \operatorname {SZRHS}_{3, 2, 4}(2, 8/\sqrt{n^{(0)}}), \\ {\varvec{\beta }}_{\text {symptom}}&\sim \operatorname {SZRHS}_{3, 2, 4}(2, 1/\sqrt{n^{(0)}}), \\ {\varvec{\beta }}_{\text {dow}}&\sim \operatorname {SZRHS}_{3, 2, 4}(2, 1/\sqrt{n^{(0)}}), \\ {\varvec{\beta }}_{\text {urban}}&\sim \operatorname {SZRHS}_{3, 2, 4}(2, 2/\sqrt{n^{(0)}}). \end{aligned}$$

Sum-to-zero multivariate normal (SZMVNormal) priors^28^ enforce identifiability by ensuring that the coefficients sum to zero. This centres the intercept $\beta _0$ as a global average, with each coefficient representing a deviation from this baseline. These priors also mitigate multicollinearity inherent in one-hot encoding without a reference category. For variable selection, we use sum-to-zero regularised horseshoe (SZRHS) priors^28^, which combine the identifiability constraint with shrinkage to induce sparsity. Further details are provided in the Supplementary Materials.

Following inference stage, variable selection is performed given a relevance threshold. We set an optimistic threshold where if the value of the median of marginal posterior of the parameter is outside the range $(-0.0513, 0.0488)$ which corresponds to a $\pm 5\%$ change from the baseline parameter $\beta _0$. Additionally, features associated with social contacts may evolve over time with the introduction or the conclusion of non-pharmaceutical interventions, vaccination, public information campaigns, and other external factors. Therefore, in this and the following analysis, we applied the model independently to waves 4 and 21 which comprised of a balanced number of first-time and repeat participants (Fig. 1), allowing us to compare the two groups at the same point in time. The final set of selected features was the union of the features selected in each wave.

### Features associated with reductions in reported contacts in association to repeat participation

Individual features associated with survey fatigue have been identified as potential concerns in several studies ^17,21^, but have not been examined in detail. We investigate associations between survey fatigue and the following variables: schooling/age (14 categories), sex (2), household size (5), employment status (9), and urban-rural typology (3) (see Table 1).

Let $Y^{(1)}_i$ denote the number of contacts reported by the *i*th repeat participant, for $i = 1,2,\ldots ,n^{(1)}$. We model $Y^{(1)}_i$ using a negative binomial distribution, $Y^{(1)} \sim \operatorname {NegBinomial}(\lambda _i, \varphi )$ $(i = 1,2,\ldots ,n^{(1)})$. The mean $\lambda _i$ is modelled on the log scale as,

2 $$\begin{aligned} \log (\lambda _i) = \hat{\beta }_0 + \underbrace{{\varvec{u}}^\top _i \hat{{\varvec{\alpha }}}}_{\text {control}} + \underbrace{{\varvec{v}}^{*\top }_i \hat{{\varvec{\beta }}}^*}_{\text {selected}} + \underbrace{ {\varvec{w}}^\top _{i,\text {age}} {\varvec{\gamma }}_{\text {age}} + {\varvec{w}}^\top _{i,\text {sex}} {\varvec{\gamma }}_{\text {sex}} + {\varvec{w}}^\top _{i,\text {hhsize}} {\varvec{\gamma }}_{\text {hhsize}} + {\varvec{w}}^\top _{i,\text {employ}} {\varvec{\gamma }}_{\text {employ}} + {\varvec{w}}^\top _{i,\text {urban}} {\varvec{\gamma }}_{\text {urban}} }_{\text {survey fatigue test features}}. \end{aligned}$$

The vector ${\varvec{u}}_i$ (dimension 19) contains one-hot encodings of the control features: schooling/age (14 categories), sex (2), and household size (3). The vector ${\varvec{v}}_i$ includes one-hot encodings of the features selected in the prior variable selection model. $\hat{\beta }_0$, $\hat{{\varvec{\alpha }}}$, and $\hat{{\varvec{\beta }}}^*$ are the posterior medians of the intercept term, the coefficients on the control features, and the coefficients corresponding to the selected test features from model (1). To test for survey fatigue effects, we include one-hot encodings ${\varvec{w}}_{i,\cdot }$ for age/schooling, sex, household size, employment status, and urban-rural typology. Let ${\varvec{\gamma }} = ({\varvec{\gamma }}_{\text {age}}, {\varvec{\gamma }}_{\text {sex}}, {\varvec{\gamma }}_{\text {hhsize}}, {\varvec{\gamma }}_{\text {employ}}, {\varvec{\gamma }}_{\text {urban}})$ denote the concatenated vector of associated coefficients. Because survey fatigue is expected to reduce reported contact intensity, we place a constrained prior on ${\varvec{\gamma }}$ that limits effects to the negative domain, ${\varvec{\gamma }} \sim \operatorname {half-RHS}^-_{3,2,4}(2,32/\sqrt{n^{(1)}})$, where $\operatorname {half-RHS}^-$ is the negative-domain variant of the regularised horseshoe prior (see Supplemental Text S1). Features were selected if the posterior median reduction in expected contact intensity exceeded 5%. We applied this model separately to survey waves 4 and 21, and used the union of selected features from both to construct the final set.

### Functional form of survey fatigue effects over the number of repeat participations

Previous studies showed that survey fatigue build up over the number of times participants contribute to consecutive survey waves ^17,19^. We leverage this empirical observation to identify a simple yet effective functional form for modelling longitudinal survey fatigue. We focused on data from waves 3-12 (May 28^th^, 2020 to July 1^st^, 2020), as these waves fell into a period of homogeneous, relaxed contact restriction measures in Germany, thereby minimising the potential confounding effects of non-pharmaceutical interventions on our analysis. We also preferred to analyse data from relatively early period of the pandemic as interventions became more heterogeneous across states with time. We analysed a total 129,831 records from 2,492 unique participants with a maximum of 11 repeats.

Let $Y_{i,r}$ denote the number of contacts reported by individual *i* in their *r*-th participation, for $i = 1,2,\ldots ,2492$ and $r = 0,1,\ldots ,11$, where $r = 0$ corresponds to first-time participation. Let $t_{i,r}$ denote the calendar date of the *r*-th participation by individual *i*. The longitudinal model is specified as:

3 $$\begin{aligned} Y_{i,r} \sim \text {NegBinomial}(\lambda _i, \varphi ), \quad \log (\lambda _i) = \beta _0 + \underbrace{{\varvec{x}}^\top _i{\varvec{\beta }}}_{\text {control}} + \underbrace{\tau (t_{i,r})}_{\text {time}} + \underbrace{\rho (r)}_{\text {fatigue}} \end{aligned}$$

The covariate vector ${\varvec{x}}_i$ includes the dummy encoding for age, sex, household size, and other relevant features identified during the previous variable selection stage. Temporal variation in contact intensity is captured by $\tau (t_{i,r})$, which represents calendar-time effects as deviations from the global intercept $\beta _0$ using a zero-mean Gaussian process characterised by the Matérn 3/2 covariance kernel

$$\begin{aligned} k(t, t') = \sigma ^2_{\tau } \left( 1 + \frac{\sqrt{3}|t - t'|}{\ell _\tau } \right) \exp \left( -\frac{\sqrt{3}|t - t'|}{\ell _\tau } \right) \end{aligned}$$

The term $\rho (r)$ captures the reduction in reported contacts associated with repeated participation, representing accumulating survey fatigue. We assessed 4 choices for modelling $\rho (r)$, which differed in the degree of assumption on the functional form of accumulating survey fatigue. To ensure the identifiability of time trends, we assumed that survey fatigue effects do not vary over calendar time and that the magnitude and shape of fatigue are consistent across participants.

The first model assumes that the reduction in reported contacts is constant regardless of the number of participations:

4 $$\begin{aligned} \rho ^{\text {Identical}}(r) = \rho \sim \operatorname {Normal}(0,1) \end{aligned}$$

We refer to this model as the *identical fixed effects* model. This is the adjustment strategy taken by Loedy et al.^21^

The second model assumes that reduction in reported contacts varies independently by the number of participations:

5 $$\begin{aligned} \rho ^{\text {Independent}}(r) = \rho _r \sim \text {Normal}(0, 1) \quad r \in \{1,2,\ldots ,R\}, \end{aligned}$$

with $\rho _0 = 0$ for identifiability. We refer to this model as the *independent fixed effects* model. This is the adjustment strategy taken by Dan et al.^19^

The third model assumes that the reduction in reported contacts is a smooth non-parametric function of the number of participations. To infer this function, we place a zero-mean Gaussian process prior with the squared exponential kernel depending on the rescaled number of participations $\tilde{r} = (r - \operatorname {mean}(r))/\operatorname {sd}(r)$

6 $$\begin{aligned} \rho ^{\text {GP}}(r)&= \rho (\tilde{r}) \sim \operatorname {GP}(0, k_{\sigma _\rho ,\ell _\rho }), \quad k_{\sigma _\rho , \ell _\rho }(\tilde{r},\tilde{r}') = \sigma ^2_\rho \exp \left( -\frac{(\tilde{r}-\tilde{r}')^2}{2 \ell _\rho ^2} \right) \nonumber \\ \sigma _{\rho }&\sim \text {inv-Gamma}(5, 5), \quad \ell _{\rho } \sim \text {inv-Gamma}(5, 5) \end{aligned}$$

for $r=1,\dotsc ,R$, and $\rho (0)=0$ to ensure identifiability. We refer to this model as the *Gaussian process* model.

For the fourth model, we take inspiration from dose-response modelling in biochemistry ^31^ and model survey fatigue with a three-parameter Hill function:

7 $$\begin{aligned} \rho ^{\text {Hill}}(r) = -\gamma \frac{e^\zeta r^\eta }{1 + e^\zeta r^\eta } \quad \text {where} \quad \gamma , \eta \in \mathbb {R}^+, \zeta \in \mathbb {R} \end{aligned}$$

where $\gamma$ is a scale parameter which control the effect size at large values of *r*, while $\zeta$ and $\eta$ in tandem controls how quickly the function approaches its minimum value. Figure S3 shows the functional form of the Hill function under different parameter values. This model is computationally more efficient than GP models, and renders, due to its parametric form, survey fatigue effects strongly predictable. Given their constraints, we assign the following priors to each parameter:

$$\begin{aligned} \gamma \sim \text {half-Normal}^+(0,1), \quad \zeta \sim \text {Normal}(0,1), \quad \eta \sim \text {Exponential}(1). \end{aligned}$$

We refer to this model as the *Hill* model.

Throughout, we used the following generic priors to the parameters in the components common across all models:

$$\begin{aligned} 1/\varphi&\sim \text {Exponential}(1), \\ \beta _0&\sim \text {Normal}(0, 5), \\ \beta _j&\sim \text {Normal}(0, 1) \quad j = 1,\ldots ,J \\ \sigma _{\tau }&{\sim } \text {inv-Gamma}(3, 1), \\ \ell _{\tau }&{\sim } \text {inv-Gamma}(5, 1). \end{aligned}$$

### Correcting for long-term survey fatigue effects with statistical contact models

Contact intensity estimates gained from statistical models fitted to data with only first-time participants avoids the bias induced by survey fatigue. However, this may mean that estimates are based on substantially smaller sample sizes (Fig. 1). Here, we describe our approach to mitigating the effects of survey fatigue. The analysis is conducted on wave 21 which contains a large number of newly recruited participants as well as repeat participants with varying numbers of prior participations.

Let $a_i$ denote the age for the *i*-th participant. The covariate vector ${\varvec{x}}_i = (x_{i1}, \ldots , x_{iP})^\top \in \{0,1\}^P$ contains the dummy encodings for sex, household size variables, and other control features identified in the preceding feature selection analysis. The vector ${\varvec{w}}_i = (w_{i1}, \ldots , w_{iQ})^\top \in \{0,1\}^Q$ includes the one-hot-encodings of features associated with survey fatigue. The contact count of participant *i* at participation *r* is modelled as:

8 $$\begin{aligned} Y_{i,r} \sim \text {NegBinomial}\left( \lambda _{i,r}, \varphi \right) , \quad \log (\lambda _{i,r}) = \beta _0 + \underbrace{f_{\sigma ,\ell }(a_i)}_{\text {age}} + \underbrace{{\varvec{x}}_i^\top {\varvec{\beta }}}_{\text {control}} + \underbrace{{\varvec{w}}^\top _i \rho (r)}_{\text {fatigue}} \end{aligned}$$

where $\beta _0$ is a global intercept, and $\rho (r) \in \mathbb {R}_{\le 0}^Q$ models the fatigue effect at participation *r* for each of the *Q* selected variables:

$$\begin{aligned} \left[\rho (r)\right]_q = -\gamma _q \exp (\zeta _q)r^{\eta _q}/(1+\exp (\zeta _q)r^{\eta _q}) \end{aligned}$$

The covariate coefficients ${\varvec{\beta }} = (\beta _1, \ldots , \beta _P)^\top$ and intercept $\beta _0$ are assigned weakly informative priors, and the inverse of the overdispersion parameter $\varphi$ is given an exponential prior:

$$\begin{aligned} \beta _0 \sim \text {Normal}(0, 5), \quad \beta _p \sim \text {Normal}(0, 1), \quad 1/\varphi \sim \text {Exp}(1). \end{aligned}$$

Let $\hat{\gamma }$, $\hat{\zeta }$, and $\hat{\eta }$ denote posterior median estimates from the longitudinal model in Eq. (3). To incorporate prior knowledge in the Bayesian framework, we assign the following weakly informative priors to the Hill function parameters:

$$\begin{aligned} \gamma _q \sim \operatorname {half-Normal}^+(\hat{\gamma }, 1), \quad \zeta _q \sim \operatorname {Normal}(\hat{\zeta }, 1), \quad \eta _q \sim \operatorname {Exponential}(\hat{\eta }), \quad \text {for } q = 1,2,\dots Q. \end{aligned}$$

The function $f_{\sigma ,\ell }(a_i): \mathbb {R} \rightarrow \mathbb {R}$ represents the non-linear effect of age on contact intensity. For computational efficiency, $f_{\sigma ,\ell }$ is modelled using a zero-mean Hilbert space approximate Gaussian process (HSGP) ^47,48^, parametrised by $\sigma , \ell$ and based on the squared exponential covariance kernel:

$$\begin{aligned} k(x, x') = \sigma ^2 \exp \left( -\frac{(x - x')^2}{2\ell ^2} \right). \end{aligned}$$

For a quantity $\theta$, let $\theta ^{(s)}$ denote its value at the *s*-th MCMC iteration, for $s = 1, \ldots , S$. Let $v_g, v_h, v_u$ and $v_j$ denote the post-stratification weights for sex, household size, urban-rural typography, and employment-status respectively, calculated as

$$\begin{aligned} v_g = \frac{P_g}{\sum _{g} P_g}, \quad v_h = \frac{P_h}{\sum _{h} P_h}, \quad v_u = \frac{P_u}{\sum _{u} P_u}, \quad v_j = \frac{n_j}{\sum _{j} n_j} \end{aligned}$$

where *P* denotes the population size estimates and *n* denotes sample sizes. We calculate the post-stratification weights for employment status from the sample as we could not obtain accurate population estimates at the level we are stratifying at. While this means that the estimates may not be completely representative of the population, it facilitates comparisons with non-model based approaches such as bootstrapping. For each MCMC sample *s*, we compute the population-weighted contact intensity as

$$\begin{aligned} \lambda ^{(s)}(a) = \sum _{g} \sum _{h} \sum _{u} \sum _{j} v_g v_h v_u v_j \exp \left( \beta _0^{(s)} + f^{(s)}_{\sigma ,\ell }(a) + \beta ^{(s)}_g + \beta ^{(s)}_h + \beta ^{(s)}_u + \beta ^{(s)}_j \right) , \end{aligned}$$

We then summarize the posterior distribution of $\lambda ^{(s)}(a)$ using the median and 95% credible interval:

9a $$\begin{aligned} \lambda _M(a)&:= \operatorname {median}\left( \{ \lambda ^{(s)}(a) \mid s = 1,\ldots , S \} \right) , \end{aligned}$$

9b $$\begin{aligned} \lambda _L(a)&:= \operatorname {quantile}_{2.5\%}\left( \{ \lambda ^{(s)}(a) \mid s = 1,\ldots , S \} \right) , \end{aligned}$$

9c $$\begin{aligned} \lambda _U(a)&:= \operatorname {quantile}_{97.5\%}\left( \{ \lambda ^{(s)}(a) \mid s = 1,\ldots , S \} \right) . \end{aligned}$$

We begin by fitting the model to the subset of data consisting only of first-time participants and treat the resulting median contact intensity, $\lambda ^0_M(a)$, as the baseline. Next, we sequentially include participants with one, two, ..., up to 20 repeat survey responses. At each step $r = 1,\ldots ,20$, we re-fit the model using all participants with at most *r* repeats and compute the preceding quantities. For comparison, we also obtained estimates in the same manner from a model without the survey fatigue adjustment term ${\varvec{w}}^\top _i \rho (r)$ in Eq. (8).

To quantify deviations from the baseline, we compute the mean absolute percentage error (MAPE) and the empirical 95% coverage of the baseline within the posterior credible intervals:

$$\begin{aligned} \operatorname {MAPE}(r) = 100 \times \frac{1}{85} \sum _{a = 0}^{84} \frac{|\lambda ^r_M(a) - \lambda ^0_M(a)|}{|\lambda ^0_M(a)|}, \quad \operatorname {Coverage}_{95\%}(r) = \frac{1}{85} \sum _{a = 0}^{84} 1\left\{ \lambda ^0_M(a) \in [\lambda ^r_L(a), \lambda ^r_U(a)] \right\} , \end{aligned}$$

where $1\{\cdot \}$ is the indicator function.

### Longitudinal and subgroup specific contact intensity estimates

To obtain survey fatigue–adjusted estimates, we applied the model defined in Eq. (8) to all 33 waves of the COVIMOD surveys as follows. Let $t = 1,2,\ldots ,33$ index the survey waves. For the first wave ($t = 1$), we used the same priors as in the previous section. From the second wave onward, we incorporated information from the previous wave by setting the priors for the individual-level feature coefficients and Hill function parameters as:

$$\begin{aligned} \beta _{t,p}&\sim \text {Normal}(\hat{\beta }_{t-1,p}, 0.3) \quad \text {for } p = 1,2,\ldots ,P, \\ \gamma _{t,q}&\sim \text {half-Normal}^+(\hat{\gamma }_{t-1,q}, 0.3), \\ \zeta _{t,q}&\sim \text {Normal}(\hat{\zeta }_{t-1,q}, 0.1), \\ \eta _{t,q}&\sim \text {half-Normal}^+\left( 1/\hat{\eta }_{t-1,q}, 0.1\right) \quad \text {for } q = 1,2,\ldots ,Q, \end{aligned}$$

where $\hat{\beta }_{t-1,p}$, $\hat{\gamma }_{t-1,q}$, $\hat{\zeta }_{t-1,q}$, and $\hat{\eta }_{t-1,q}$ denote the posterior means from wave $t-1$. This sequential fitting procedure allows the model to leverage prior information to partially resolve the identifiability issue between $\beta _{t,p}$ and the Hill function parameters when no first-time participants are present.

For each MCMC sample *s*, we compute the population-weighted contact intensity as:

10 $$\begin{aligned} \lambda ^{(s)} = \sum _a \sum _g \sum _h \sum _j v_{a,g} v_h v_u v_j \exp \left( \beta _0^{(s)} + f^{(s)}_{\sigma ,\ell }(a) + \beta ^{(s)}_g + \beta ^{(s)}_h + \beta ^{(s)}_{u} + \beta ^{(s)}_j \right) , \end{aligned}$$

where $v_{a,g} = P_{a,g} / \sum _a \sum _g P_{a,g}$ and $v_h, v_u, v_j$ is defined as in the previous section. Subgroup-specific contact intensities are obtained by including additional covariates and restricting the summation to relevant subpopulations. For example, the contact intensity among stay-at-home parents aged 19–64 is computed as:

$$\begin{aligned} \lambda _{\text {stay-at-home, 19--64}}^{(s)} = \sum _{a=19}^{64} \sum _g \sum _h \sum _u\tilde{v}_{a,g} v_h v_u \exp \left( \beta _0^{(s)} + f^{(s)}_{\sigma ,\ell }(a) + \beta ^{(s)}_g + \beta ^{(s)}_h + \beta ^{(s)}_{u} + \beta ^{(s)}_{\text {stay-at-home}} \right) , \end{aligned}$$

where the weights $\tilde{v}_{a,g}$ are normalized to sum to 1 within the 19–64 age range:

$$\begin{aligned} \tilde{v}_{a,g} = \frac{P_{a,g}}{\sum _{a=19}^{64} \sum _g P_{a,g}}. \end{aligned}$$

We summarize the posterior distribution of $\lambda ^{(s)}$ using the median, 2.5% quantile, and 97.5% quantile, as in Eq. (9a-c). The resulting estimates were compared against (i) those from a simple bootstrap procedure ^25^, (ii) model-based estimates without the survey fatigue adjustment term ${\varvec{w}}^\top _i \rho (r)$, and (iii) estimates based on data from first-time participants only, in waves with at least 300 such participants.

## Supplementary Information

Supplementary Information.

## Data Availability

The data required to reproduce the results of this work is publicly available from Zenodo. All the code required to reproduce the results is publicly available from Github.
