# Supplementary material for: Addressing survey fatigue bias in longitudinal social contact studies to improve pandemic preparedness
Source: Sci Rep. 2025 May 23;15:17935. doi: 10.1038/s41598-025-02235-0 (PMC12102313; doi:10.1038/s41598-025-02235-0)
Supplement: Supplementary file 1 — Supplementary Information. [file 41598_2025_2235_MOESM1_ESM.pdf]

Supplementary Materials

Shozen Dan[1,*] **and Zhi Ling**[1,2]

[1]Imperial College London, Department of Mathematics, United Kingdom
[2]National University of Singapore, Saw Swee Hock School of Public Health, Singapore
[*]shozen.dan21@imperial.ac.uk

Horseshoe prior

The horseshoe (HS) prior proposed by Carvalho et al.[1,2] is a sparsity inducing prior that falls within the global-local shrinkage framework. Suppose we have parameters θ_j for $j = 1,2\dots,m$. The prior has a hierarchical structure where

$$\theta_j \sim \text{Normal}(0, \tau^2\lambda_j^2), \quad \tau \sim \text{Cauchy}^+(0,1), \quad \lambda_j \sim \text{Cauchy}^+(0,1). \tag{1}$$

The global shrinkage parameter τ pulls the posterior of the parameters toward zero while the local shrinkage parameter λ_i allows parameters with strong evidence against the null to escape shrinkage. Suppose that the observed data $y_j|\theta_j \sim \text{Normal}(\theta_j, \sigma^2)$. Conditional on σ, τ, λ_j and the data, the posterior expectation of θ_j has the form

$$\mathbb{E}[\theta_j|y_j,\sigma,\tau,\lambda_j] = \frac{\sigma^2\tau^2\lambda_j^2}{\tau^2\lambda_j^2+\sigma^2}y_j.$$

When $\sigma = \tau = 1$, then the expression above simplifies to

$$\mathbb{E}[\theta_j|y_j,\lambda_j] = \frac{\lambda_j^2}{1+\lambda_j^2}y_j = (1-\kappa_j)y_j$$

where $\kappa_j = 1/(1+\lambda_j^2)$. By further integrating with respect to κ_j, we have that

$$\mathbb{E}[\theta_j|y_j] = \int_0^1 (1-\kappa_j)y_j p(\kappa_j|\boldsymbol{y})d\kappa_j = (1-\mathbb{E}[\kappa_j|\boldsymbol{y}])y_j.$$

Hence, we may interpret κ_j as a factor that controls the shrinkage towards 0. Among the many choices of priors for λ_j, specifying a standard half-Cauchy prior implies a $\text{Beta}(1/2,1/2)$ prior on κ_j which resembles a horse's shoe, giving the prior its distinctive name.

Horseshoe prior for linear regression

Suppose we are working in a linear regression framework where

$$y_i = \boldsymbol{\beta}^\top \boldsymbol{x}_i + \varepsilon_i, \quad \varepsilon_i \sim \text{Normal}(0,\sigma^2), \quad 1,\dots,n.$$

Here, $\boldsymbol{x}_i$ is a P-dimensional vector of features, $\boldsymbol{\beta}$ is a P-dimensional vector of coefficients, and σ^2 is the sampling variance. Suppose that our goal is to infer a sparse vector for $\boldsymbol{\beta}$, where only features with a strong correlation with the outcome y can have coefficients with significant posterior density away from zero. To this end, we may employ a horseshoe prior as the prior on $\boldsymbol{\beta}$. Note that if an intercept were to be included in the model, then it is given a relatively flat prior with no shrinkage.

For the normal model, the full-conditional distribution of $\boldsymbol{\beta}$ is a multivariate normal distribution with mean and coviarance

$$\bar{\boldsymbol{\beta}} = \tau^2\boldsymbol{\Lambda}\left(\tau^2\boldsymbol{\Lambda}+\sigma^2(\boldsymbol{X}^\top\boldsymbol{X})^{-1}\right)\hat{\boldsymbol{\beta}}, \quad \boldsymbol{\Sigma} = \left(\tau^{-2}\boldsymbol{\Lambda}^{-1}+\frac{1}{\sigma^2}\boldsymbol{X}^\top\boldsymbol{X}\right)^{-1}$$

where $\boldsymbol{\Lambda} = \text{diag}(\lambda_1^2,\dots,\lambda_P^2)$ and $\hat{\boldsymbol{\beta}} = (\boldsymbol{X}^\top\boldsymbol{X})^{-1}\boldsymbol{X}^\top\boldsymbol{y}$ is the ordinary least squares estimate. If we assume that the features are uncorrelated with zero mean and variances $\mathbb{V}[X_j] = s_j^2$, then $\boldsymbol{X}^\top\boldsymbol{X} \approx \text{diag}(s_1^2,\dots,s_P^2)$. This allows us to approximate the posterior of the coefficients as follows:

$$\bar{\beta}_j = (1-\kappa_j)\hat{\beta}_j, \quad \kappa_j = \frac{1}{1+n\sigma^{-2}\tau^2 s_j^2\lambda_j^2} = \frac{1}{1+a_j}$$

where $a_j = \sqrt{n}\sigma^{-1}\tau s_j$. By employing independent standard half-Cauchy priors for λ_j, the shrinkage factor κ_j has the prior

$$p(\kappa_j|\tau,\sigma) = \frac{1}{\pi}\frac{a_j}{(a_j^2-1)\kappa_j+1}\frac{1}{\sqrt{\kappa_j(1-\kappa_j)}}$$

where the mean an variance can be shown to be,

$$\mathbb{E}[\kappa_j|\tau,\sigma] = \frac{1}{1+a_j}, \quad \mathbb{V}[\kappa_j|\tau,\sigma] = \frac{a_j}{2(1+a_j)^2}.$$

Piironen and Vehtari[3] defines the effective number of non-zero coefficients as

$$m_{\text{eff}} = \sum_j^P (1-\kappa_j).$$

If we place the additional assumption that each regression feature has a unit variance $s_j^2 = 1$, which is not unreasonable as we can always standardise the values beforehand, the expectation of the effective number of non-zero coefficient is

$$\mathbb{E}[m_{\text{eff}}|\tau,\sigma] = \frac{\tau\sigma^{-1}\sqrt{n}}{1+\tau\sigma^{-1}\sqrt{n}}P.$$

This last expression is helpful in guiding us to choose a specific value for the global scale parameter τ. Suppose that we have prior knowledge that the number of non-zero coefficients are likely to be $0 < p_0 < P$, then substituting p_0 for the LHS and solving for τ gives us

$$\tau_0 = \frac{p_0}{P-p_0}\frac{\sigma}{\sqrt{n}}.$$

Regularised horseshoe prior

The horseshoe prior suffers from a lack of consensus in how to carry out the inference for τ, which controls the overall sparsity in the parameter vector, and that parameters far from zero are not regularised. To enable sensible inference on τ and to ensure that large coefficients are slightly regularised, Piironen and Vehtari[3] proposed the regularised horseshoe (RHS) prior which modifies the local shrinkage parameter λ_j:

$$\theta_j|\lambda_j,\tau,c \sim \text{Normal}(0,\tau^2\tilde{\lambda}_j^2), \quad \tilde{\lambda}_j^2 = \frac{c^2\lambda_j^2}{c^2+\tau^2\lambda_j^2}$$

where the priors on τ and λ_j remain as is. Unless substantial information about the scale of the coefficients is the available, one can assume $c^2 \sim \text{Inv-Gamma}(\nu/2, \nu s^2/2)$.

In addition to the problem of vanishing means, the original horseshoe prior suffers from sampling issues in Markov chain Monte Carlo (MCMC) methods such as Stan. This is caused by the posterior having an extreme funnel shape and related to the heavy tails of the Cauchy distribution. To mitigate this issue, we follow recommendations by Piironen and Vehtari[3] and replace the standard half-Cauchy priors with half-student-t priors with a small degree of freedom, *e.g.* $\nu = 3$. In summary, we implement the RHS prior in Stan as follows:

$$\begin{aligned}
\theta_j &\sim \text{Normal}(0,\tau^2\tilde{\lambda}_j^2), \quad \tilde{\lambda}_j^2 = \frac{c^2\tilde{\lambda}_j^2}{c^2+\tau^2\lambda_j^2} \\
\tau &\sim \text{student-}t^+_{\nu_1}(0,\tau_0^2) \\
c^2 &\sim \text{inv-Gamma}(\nu_2/2, \nu_2 s^2/2) \\
\lambda_j &\sim \text{student-}t^+_{\nu_3}(0,1)
\end{aligned}$$

and denote this composite prior with the short-hand $\text{RHS}_{\nu_1,\nu_2,\nu_3}(s^2,\tau_0)$ to reduce notational clutter.

Construction of sum-to-zero priors

In this section, we provide details on the theory and implementation of Bayesian shrinkage priors subject to sum-to-zero constraints. We note that this is a specific application of the work by Ling and Dan[4] and refer readers to their work for descriptions of a more general framework.

The horseshoe prior for linear regression described in Piironen and Vehtari[5] assumes that features are continuous and independent. However, we often have to work with discrete categorical features such as sex and occupation. The standard approach for handling categorical features in linear regression is to encode the categories as dummy feature(s) where the feature takes value 1 if the observation belongs to that category and 0 otherwise. In doing so, we have to set a reference category in order to avoid multi-collinearity and identifiability issues. Consequently, if there were n observations of a feature with J categories, then the resulting dummy matrix will be of dimension $n \times (J-1)$. This is problematic for variable selection because the relevance of that feature is evaluated with respect to the reference category. If the reference category is changed, then a different set of variables may be chosen.

Ideally, we would like the model to determine if the effect of a feature is significant with respect to a global baseline. For instance, consider the following model that estimates the effect of sex on a outcome variable y:

$$y_i = \beta_0 + \beta_{\text{men}} 1\{i \text{ is a man}\} + \beta_{\text{women}} 1\{i \text{ is a women}\} + \varepsilon_i, \quad \varepsilon_i \sim \text{Normal}(0, \sigma^2), \quad i = 1, \dots, n. \tag{2}$$

The quantities $\mu_{\text{men}} = \beta_0 + \beta_{\text{men}}$ and $\mu_{\text{women}} = \beta_0 + \beta_{\text{women}}$ should represent the average value of y for men and women, respectively. If we want β_0 to represent the global average of y, and if we want β_{men} and β_{women} to act as offsets to this mean, then the coefficient should satisfy the constraint $\beta_{\text{man}} + \beta_{\text{women}} = 0$. In general, if we have a categorical feature with J-categories, we would like the coefficient vector $\boldsymbol{\beta} = (\beta_1, \dots, \beta_J)^\top$ to satisfy $\sum_{j=1}^{J} \beta_j = 0$ or equivalently $\mathbf{1}^\top \boldsymbol{\beta} = 0$ where $\mathbf{1} = (1, \dots, 1)^\top$ is a J-dimensional vector of ones.

Here, we give a brief description of how to construct and sample from a prior that satisfies this sum-to-zero constraint. Let $\boldsymbol{\Sigma}$ be a $J \times J$ covariance matrix defined as

$$\boldsymbol{\Sigma} = \boldsymbol{I} - \frac{1}{J}\mathbf{1}\mathbf{1}^\top$$

where $\boldsymbol{I}$ is a $J \times J$ identity matrix. Then, the coefficient vector $\boldsymbol{\beta}$ sampled from $\text{MVNormal}\big(\mathbf{0}, \sigma^2 \boldsymbol{\Sigma}\big)$ has elements which sum to 0 with probability 1.

Proof. Let $Y = \mathbf{1}^\top \boldsymbol{\beta} = \sum_{j=1}^{J} \beta_j$ where $\boldsymbol{\beta} \sim \text{MVNormal}(\mathbf{0}, \sigma^2 \boldsymbol{\Sigma})$ and $\boldsymbol{\Sigma} = \boldsymbol{I} - \frac{1}{J}\mathbf{1}\mathbf{1}^\top$. Then,

$$\mathbb{E}[Y] = \mathbf{1}^\top \mathbb{E}[\boldsymbol{\beta}] = 0,$$
$$\mathbb{V}[Y] = \mathbf{1}^\top \mathbb{V}[\boldsymbol{\beta}] \mathbf{1} = \sigma^2 \mathbf{1}^\top \left(\boldsymbol{I} - \frac{1}{J}\mathbf{1}\mathbf{1}^\top \right) \mathbf{1} = \sigma^2 \left(\mathbf{1}^\top \mathbf{1} - \frac{1}{J} \mathbf{1}^\top \mathbf{1}\mathbf{1}^\top \mathbf{1} \right) = \sigma^2 (J - \frac{1}{J} J^2) = 0.$$

This implies that $\mathbb{E}[Y^2] = \mathbb{V}[Y] + (\mathbb{E}[Y])^2 = 0$ and because $Y^2 \geq 0$, we have $Y^2 = 0$ almost surely, which implies $Y = 0$ almost surely. □

If we chose $\sigma^2 = J/(J-1)$, the diagonals of the covariance matrix takes value 1 which implies that marginal distributions of $\boldsymbol{\beta}$ are standard normal distributions, *i.e.*, $\beta_j \sim \text{Normal}(0, 1)$.

To perform inference using probabilistic programming languages such as Stan, we must sample from the target distribution. However, since the covariance matrix $\boldsymbol{\Sigma}$ is rank-deficient (and thus singular), standard multivariate normal samplers cannot be used directly. Let $\boldsymbol{M}$ be any $J \times (J-1)$ matrix that maps vectors in $\mathbb{R}^{J-1}$ to the sum-to-zero subspace of $\mathbb{R}^J$. For example, one may choose

$$\boldsymbol{M} := \begin{pmatrix} 1 & 0 & \cdots & 0 \\ 0 & 1 & \cdots & 0 \\ \vdots & \vdots & \ddots & \vdots \\ 0 & 0 & \cdots & 1 \\ -1 & -1 & \cdots & -1 \end{pmatrix}.$$

Applying the Gram–Schmidt process to $\boldsymbol{M}$ yields the factorization $\boldsymbol{M} = \boldsymbol{Q}\boldsymbol{R}$, where $\boldsymbol{Q}$ is an orthonormal basis for the sum-to-zero subspace. Define an auxiliary parameter vector $\boldsymbol{\gamma} \in \mathbb{R}^{J-1}$ by $\boldsymbol{\beta} = \boldsymbol{Q}\boldsymbol{\gamma}$. Since $\boldsymbol{Q}^\top \boldsymbol{Q} = \boldsymbol{I}$, it follows that $\boldsymbol{\gamma} = \boldsymbol{Q}^\top \boldsymbol{\beta}$. The mean and covariance of $\boldsymbol{\gamma}$ are then

$$\mathbb{E}[\boldsymbol{\gamma}] = \mathbb{E}[\boldsymbol{Q}\boldsymbol{\beta}] = \mathbf{0},$$
$$\mathbb{V}[\boldsymbol{\gamma}] = \mathbb{V}[\boldsymbol{Q}^\top \boldsymbol{\beta}] = \boldsymbol{Q}^\top \mathbb{V}[\boldsymbol{\beta}] \boldsymbol{Q} = \sigma^2 \boldsymbol{Q}^\top \boldsymbol{\Sigma} \boldsymbol{Q} = \sigma^2 \boldsymbol{Q}^\top \left(\boldsymbol{I} - \frac{1}{J}\mathbf{1}\mathbf{1}^\top \right) \boldsymbol{Q} = \tilde{\boldsymbol{\Sigma}}.$$

Proof that $\tilde{\boldsymbol{\Sigma}}$ is positive semi-definite. Let $\boldsymbol{x} \in \mathbb{R}^{J-1}$ be any non-zero vector and define $\boldsymbol{y} = \boldsymbol{Q}\boldsymbol{x}$. Then,

$$\boldsymbol{x}^\top \tilde{\boldsymbol{\Sigma}} \boldsymbol{x} = \sigma^2 \boldsymbol{x}^\top \boldsymbol{Q}^\top \Big(\boldsymbol{I} - \frac{1}{J}\mathbf{1}\mathbf{1}^\top\Big)\boldsymbol{Q}\boldsymbol{x} = \sigma^2 \boldsymbol{y}^\top \Big(\boldsymbol{I} - \frac{1}{J}\mathbf{1}\mathbf{1}^\top\Big)\boldsymbol{y}.$$

Since $\boldsymbol{y}$ lies in the sum-to-zero subspace (i.e., $\mathbf{1}^\top \boldsymbol{y} = 0$), we have

$$\boldsymbol{y}^\top \Big(\boldsymbol{I} - \frac{1}{J}\mathbf{1}\mathbf{1}^\top\Big)\boldsymbol{y} = \boldsymbol{y}^\top \boldsymbol{y} \geq 0.$$

Thus, $\tilde{\boldsymbol{\Sigma}}$ is positive semi-definite. □

Because $\tilde{\boldsymbol{\Sigma}}$ is positive semi-definite, we can apply the Cholesky decomposition, $\tilde{\boldsymbol{\Sigma}} = \boldsymbol{L}\boldsymbol{L}^\top$. To sample $\boldsymbol{\beta}$, we first draw $\boldsymbol{z} \sim \text{MVNormal}(\mathbf{0}, \boldsymbol{I}_{J-1})$, and then compute

$$\boldsymbol{\beta} = \boldsymbol{Q}\boldsymbol{\gamma} = \boldsymbol{Q}(\boldsymbol{L}\boldsymbol{z}).$$

We denote this prior by $\boldsymbol{\beta} \sim \text{SZMVNormal}(\sigma^2)$.

Sum-to-zero horseshoe prior

To extend the sum-to-zero multivariate normal prior to include horseshoe shrinkage effects, define the matrix

$$\boldsymbol{\Sigma}_{\boldsymbol{\Lambda}} = \boldsymbol{\Lambda} - \boldsymbol{\Lambda}\mathbf{1}(\mathbf{1}^\top \boldsymbol{\Lambda}\mathbf{1})^{-1}\mathbf{1}^\top \boldsymbol{\Lambda}.$$

where $\boldsymbol{\Lambda} = \text{diag}(\lambda_1^2, \ldots, \lambda_J^2)$ is the diagonal matrix containing the local shrinkage parameters of the horseshoe prior. The diagonal elements of this matrix is computed as

$$[\boldsymbol{\Sigma}_{\boldsymbol{\Lambda}}]_{jj} = \lambda_j^2 - \frac{\lambda_j^4}{\sum_{k=1}^J \lambda_k^2} = \lambda_j^2 \left(1 - \frac{\lambda_j^2}{\sum_{k=1}^J \lambda_k^2}\right)$$

If we assume a priori that $\lambda_j^2 / \sum_{k=1}^J \lambda_k^2 = 1/J$, then the marginal of a vector of random variables distributed according to

$$\boldsymbol{\beta} | \tau^2, \boldsymbol{\Lambda} \sim \text{MVNormal}\left(\mathbf{0}, \frac{J}{J-1}\tau^2 \boldsymbol{\Sigma}_{\boldsymbol{\Lambda}}\right)$$

is distributed according to a normal distribution with variance $\tau^2 \lambda_j^2$.

Proof that $\boldsymbol{Q}^\top \boldsymbol{\Sigma}_{\boldsymbol{\Lambda}} \boldsymbol{Q}$ is positive semi-definite. Let $\boldsymbol{x} \in \mathbb{R}^{J-1}$ be any non-zero vector and define $\boldsymbol{y} = \boldsymbol{Q}\boldsymbol{x}$. Then,

$$\boldsymbol{x}^\top \boldsymbol{\Sigma}_{\boldsymbol{\Lambda}} \boldsymbol{x} = \boldsymbol{x}^\top \boldsymbol{Q}^\top \Big(\boldsymbol{\Lambda} - \boldsymbol{\Lambda}\mathbf{1}(\mathbf{1}^\top \boldsymbol{\Lambda}\mathbf{1})^{-1}\mathbf{1}^\top \boldsymbol{\Lambda}\Big)\boldsymbol{Q}\boldsymbol{x} = \boldsymbol{y}^\top \Big(\boldsymbol{\Lambda} - \boldsymbol{\Lambda}\mathbf{1}(\mathbf{1}^\top \boldsymbol{\Lambda}\mathbf{1})^{-1}\mathbf{1}^\top \boldsymbol{\Lambda}\Big) = \boldsymbol{y}^\top \boldsymbol{\Lambda}\boldsymbol{y} - \boldsymbol{y}^\top \boldsymbol{\Lambda}\mathbf{1}(\mathbf{1}^\top \boldsymbol{\Lambda}\mathbf{1})^{-1}\mathbf{1}^\top \boldsymbol{\Lambda}\boldsymbol{y}.$$

which evaluates to

$$\sum_{j=1}^J \lambda_j^2 y_j^2 - \left(\sum_{j=1}^J \lambda_j^2 y_j\right)^2 / \sum_{j=1}^J \lambda_j^2.$$

By the Cauchy–Schwarz inequality, we have

$$\left(\sum_{j=1}^J \lambda_j^2 y_j\right)^2 \leq \left(\sum_{j=1}^J \lambda_j^2\right)\left(\sum_{j=1}^J \lambda_j^2 y_j^2\right).$$

Dividing both sides by $\sum_{j=1}^J \lambda_j^2$ yields

$$\frac{\left(\sum_{j=1}^J \lambda_j^2 y_j\right)^2}{\sum_{j=1}^J \lambda_j^2} \leq \sum_{j=1}^J \lambda_j^2 y_j^2.$$

This implies that $\boldsymbol{x}^\top \boldsymbol{\Sigma}_{\boldsymbol{\Lambda}} \boldsymbol{x} \geq 0$ and thus completes the proof. □

Since $\boldsymbol{Q}^\top \boldsymbol{\Sigma}_{\boldsymbol{\Lambda}} \boldsymbol{Q}$ is a positive semi-definite matrix, we follow the same approach used to sample from sum-to-zero multivariate normal priors to sample from this prior. One may replace λ_j^2 with the regularized version $\tilde{\lambda}_j^2$. We denote this prior with the shorthand $\boldsymbol{\beta} \sim \text{SZRHS}_{\nu1,\nu2,\nu3}(s^2, \tau_0)$.

Half horseshoe prior

To incorporate our belief that certain parameters must lie in either the positive or negative real domain, we can constrain the horseshoe and regularized horseshoe priors accordingly. Here we describe how to constrain the horseshoe prior to the positive domain (the negative case is analogous). Recall that under the standard horseshoe prior,

$$\mathbb{E}[\beta_j \mid \tau, \lambda_j] = 0 \quad \text{and} \quad \mathbb{V}[\beta_j \mid \tau, \lambda_j] = \tau^2 \lambda_j^2.$$

A common sampling strategy is to first draw an auxiliary variable $z_j \sim \text{Normal}(0,1)$ and then set $\beta_j = \tau \lambda_j z_j$.

To restrict β_j to the positive real domain, we instead sample $z_j \sim \text{half-Normal}^+(0, \sigma^2)$ and compute β_j in the same way. When $\sigma^2 = 1$, one can show that

$$\mathbb{E}[\beta_j \mid \tau, \lambda_j] = \sqrt{\frac{2}{\pi}} \tau \lambda_j \quad \text{and} \quad \mathbb{V}[\beta_j \mid \tau, \lambda_j] = \left(1 - \frac{2}{\pi}\right) \tau^2 \lambda_j^2.$$

Although this constrained prior has a non-zero mean, note that $\mathbb{E}[\beta_j \mid \tau, \lambda_j] \to 0$ as $\tau \to 0$, that is, the global shrinkage parameter τ still pulls β_j toward zero. To match the variance of the original horseshoe prior, we adjust the variance of z_j by setting $\sigma^2 = \left(1 - \frac{2}{\pi}\right)^{-1}$. Then,

$$\mathbb{V}[\beta_j \mid \tau, \lambda_j] = \left(1 - \frac{2}{\pi}\right) \left(1 - \frac{2}{\pi}\right)^{-1} \tau^2 \lambda_j^2 = \tau^2 \lambda_j^2.$$

One may replace λ_j^2 with the regularised $\tilde{\lambda}_j^2$ to facilitate sampling in probabilistic programming languages and to incorporate prior knowledge. For succinctness, we will denote this prior as $\text{half-RHS}_{\nu_1,\nu_2,\nu_3}(s^2, \tau_0)$.

References

1. Carvalho, C. M., Polson, N. G. & Scott, J. G. Handling Sparsity via the Horseshoe. In *Proceedings of the Twelfth International Conference on Artificial Intelligence and Statistics*, 73–80 (PMLR, 2009). ISSN: 1938-7228.
2. Carvalho, C. M., Polson, N. G. & Scott, J. G. The horseshoe estimator for sparse signals. *Biometrika* **97**, 465–480, DOI: 10.1093/biomet/asq017 (2010).
3. Piironen, J. & Vehtari, A. Sparsity information and regularization in the horseshoe and other shrinkage priors. *Electron. J. Stat.* **11**, 5018–5051, DOI: 10.1214/17-EJS1337SI (2017). Publisher: Institute of Mathematical Statistics and Bernoulli Society.
4. Ling, Z. & Dan, S. Bayesian shrinkage priors subject to linear constraints, DOI: 10.48550/arXiv.2504.09052 (2025).
5. Piironen, J., Paasiniemi, M. & Vehtari, A. Projective inference in high-dimensional problems: Prediction and feature selection. *Electron. J. Stat.* **14**, DOI: 10.1214/20-EJS1711 (2020).
6. Funk, S. *et al.* socialmixr: Social Mixing Matrices for Infectious Disease Modelling (2024).

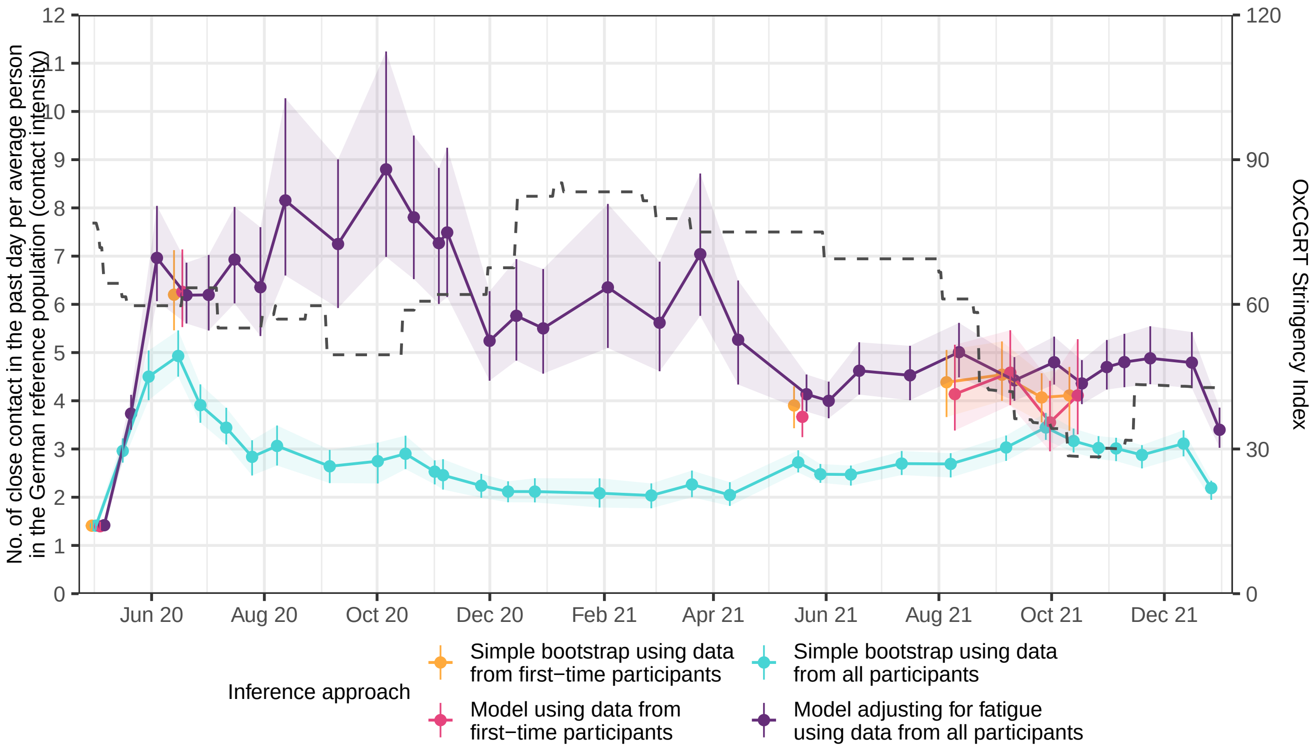

Figure S1. Comparison between simple bootstrap based and Bayesian model based estimates for longitudinal contact intensity during the COVID-19 pandemic in Germany., national-level contact intensity estimates (point: simple bootstrap mean or posterior median estimate, linerange: 95% bootstrap confidence or 95% credible intervals) are shown according to different estimation approaches: Simple bootstrap[6] using data from first-time participants only, for waves with more than 300 first-time participants (orange); Simple bootstrap[6] using data from all participants and not adjusting for reporting fatigue (blue); Bayesian model using data from first-time participants only, for waves with more than 300 first-time participants (pink); Bayesian model using data from all participants and adjusting for reporting fatigue (purple). The dashed line represents the OxCGRT Stringency Index with higher values indicating a higher degree of contact restrictions (min: 0, max: 100).

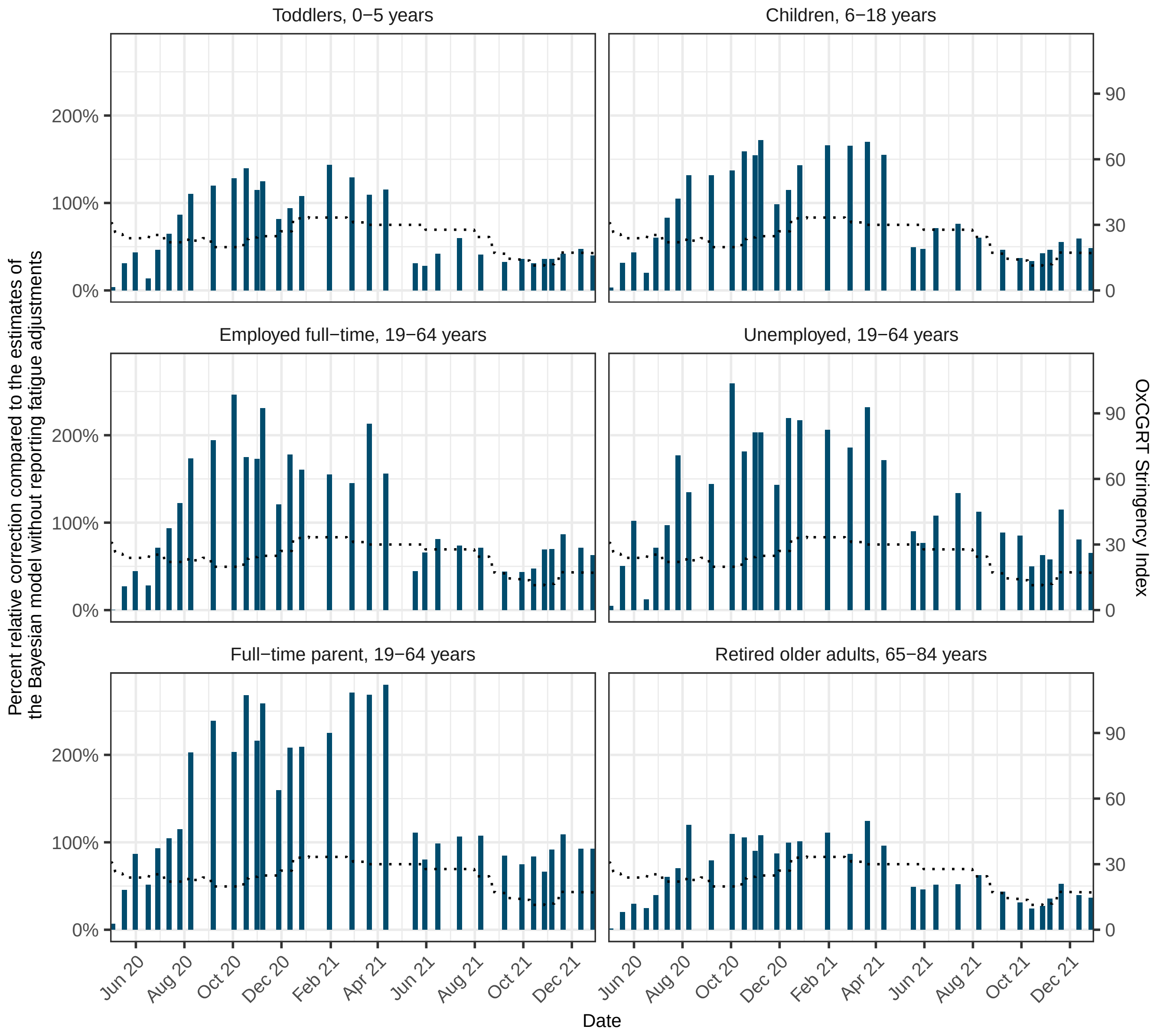

Figure S2. Percent relative correction of contact intensity estimates from the Hill model against the Bayesian model without reporting fatigue adjustments. Blue bars represent percent change in median contact intensity estimates from the Hill model relative to estimates from the Bayesian fatigue un-adjusted model. The dotted lines represents the OxCGRT Stringency Index with higher values indicating a higher degree of contact restrictions (min: 0, max: 100).

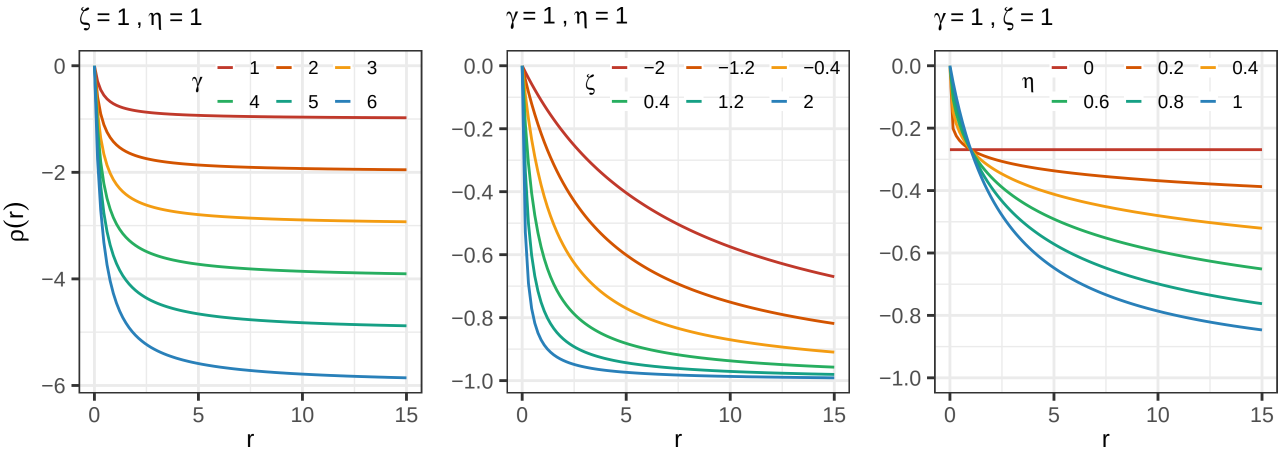

Figure S3. **Functional form of the Hill function under different parameter values.** Left: varying the scale parameter γ from 1 to 6 while fixing shape parameters ζ and η at 1. Centre: varying the shape parameter ζ from -2 to 2 while fixing the scale parameter γ and the second shape parameter η at 1. Right: varying the second shape parameter η from 0 to 1 while fixing the scale parameter γ and the first shape parameter ζ at 1.
